# MXenes as emerging materials to repair electroactive tissues and organs

**DOI:** 10.1016/j.bioactmat.2025.01.035

**Published:** 2025-03-03

**Authors:** Keshav Narayan Alagarsamy, Leena Regi Saleth, Saravanan Sekaran, Laura Fusco, Lucia Gemma Delogu, Maksym Pogorielov, Açelya Yilmazer, Sanjiv Dhingra

**Affiliations:** a Institute of Cardiovascular Sciences, St. Boniface Hospital Albrechtsen Research Centre, Department of Physiology and Pathophysiology, Max Rady College of Medicine, Rady Faculty of Health Sciences, Biomedical Engineering Program, University of Manitoba, Winnipeg, Manitoba, R2H 2A6, Canada; b Department of Prosthodontics, Saveetha Dental College and Hospitals, Saveetha Institute for Medical and Technical Sciences, Saveetha University, Chennai, 600077, Tamil Nadu, India; c University of Science & Technology, Abu Dhabi, United Arab Emirates; d ImmuneNano-Lab, Department of Biomedical Sciences, University of Padua, Padua, Italy; e Sumy State University, 2 Rymskogo-Korsakova Street, Sumy, 40007, Ukraine; f University of Latvia, 3 Jelgavas Street, Riga, LV-1004, Latvia; g Department of Biomedical Engineering, Ankara University, Golbasi, Ankara, 06830, Turkey; h Stem Cell Institute, Ankara University, Balgat, Ankara, 06520, Turkey

**Keywords:** MXenes, Biosensors, Tissue engineering, Electroactive

## Abstract

Nanomaterials with electroactive properties have taken a big leap for tissue repair and regeneration due to their unique physiochemical properties and biocompatibility. MXenes, an emerging class of electroactive materials have generated considerable interest for their biomedical applications from bench to bedside. Recently, the application of these two-dimensional wonder materials have been extensively investigated in the areas of biosensors, bioimaging and repair of electroactive organs, owing to their outstanding electromechanical properties, photothermal capabilities, hydrophilicity, and flexibility. The currently available data reports that there is significant potential to employ MXene nanomaterials for repair, regeneration and functioning of electroactive tissues and organs such as brain, spinal cord, heart, bone, skeletal muscle and skin. The current review is the first report that compiles the most recent advances in the application of MXenes in bioelectronics and the development of biomimetic scaffolds for repair, regeneration and functioning of electroactive tissues and organs including heart, nervous system, skin, bone and skeletal muscle. The content in this article focuses on unique features of MXenes, synthesis process, with emphasis on MXene-based electroactive tissue engineering constructs, biosensors and wearable biointerfaces. Additionally, a section on the future of MXenes is presented with a focus on the clinical applications of MXenes.

## Highlights

• This review compiles latest advancements in the use of MXenes for the repair of electroactive tissues and organs.
• Synthesis processes and unique electromechanical properties of MXene-based biomimetic scaffolds and wearable biointerfaces.
• The paper systematically discusses the applications of MXenes in biosensors, bioimaging, and electroactive tissue engineering for heart, brain, spinal cord, skin, bone, and muscle repair.
• Future directions and clinical potential of MXenes in bioelectronics and regenerative medicine, from bench to bedside.

## Graphical abstract

**Figure:**
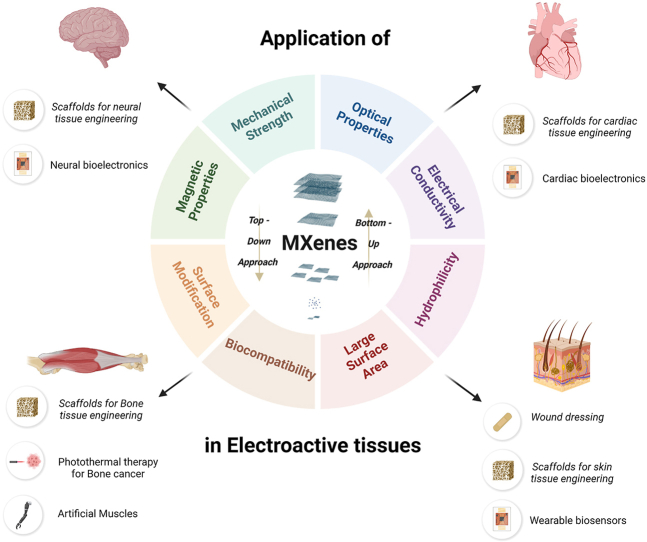

## 1 Introduction

**Fig. 1:**
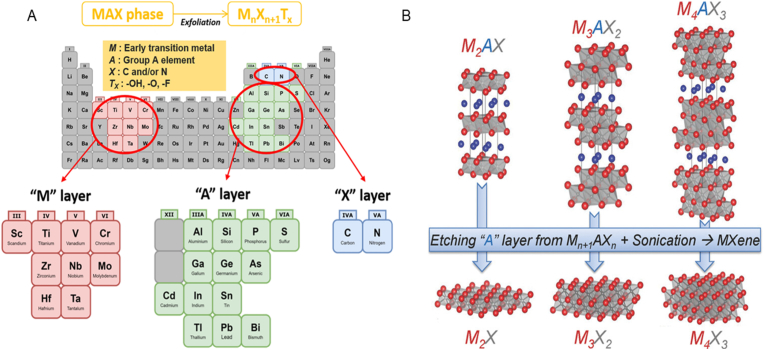
(A) Elemental composition of MXene and its bulk MAX phase. This figure is reproduced with permission from Ref. [14]. Copyright © 2021, Springer Nature, The Author(s). (B) The different crystal structures of MAX phase and its corresponding MXenes, based on the number of M layers separating the A layers. This figure is reproduced with permission from Ref. [10]. © 2013 WILEY‐VCH Verlag GmbH & Co. KGaA, Weinheim.

Recent progress in the field of biomaterials synthesis and application has revolutionized the fields of medical biotechnology and tissue engineering. A biomaterial is any material that can interact with cells and tissues and can be used to treat, diagnose, or augment body functions over an extended period [1]. Over the years, biomaterials are constructed based on their application, either from natural polymers or synthetic materials such as metals or ceramics, or as a composite of natural and synthetic materials [2]. Several biomaterials have been employed for biomedical applications because of their unique ability to be tailored for specific functions. In recent years, 2 dimensional (2D) materials have become popular in the field of tissue engineering due to their remarkable physicochemical properties. The recent research is extensively centered toward developing 2D materials such as graphene and its derivatives [3], hexagonal boron nitride (h-BN) [4], layered double hydroxides [5] and transition metal oxides (TMO) for biomedical applications [6]. More recently MXenes [7] have received significant attention for their application in the field of medical technology. MXenes represent a new and exciting family of materials, derived from the etching of MAX phases, which consist of transition metal carbides, nitrides, and carbonitrides. Since their introduction, scientists have developed over 150 different MAX phases, and more than 30 distinct MXenes have been synthesized and thoroughly studied [8]. The typical chemical structure of MXenes is expressed as M_(n+1)_X_n_, where n can range from 1 to 3. In this formula, "M" stands for a transition metal, such as titanium (Ti), tantalum (Ta), niobium (Nb), chromium (Cr), molybdenum (Mo), zirconium (Zr), hafnium (Hf), or vanadium (V), while "X" refers to either carbon (C) or nitrogen (N) [9]. It can be deduced that these materials can be derived from their respective parent MAX phases via selective etching of 'A' followed by delamination to produce MXenes that can be multilayered or single-layered (Fig. 1A). Etching the MAX phase results in the formation of several surface functional groups on MXenes, such as hydroxyl, oxygen and fluorine groups. These surface terminations play a significant role in modifying the properties of the MXene material. These surface terminations are represented by "T" which modified the structural formula from M_(n+1)_X_n_ to M_(n+1)_X_n_T_x_, where "T" accounts for the different chemical groups attached to the MXene surface after synthesis [10] (Fig. 1A).

**Fig. 2:**
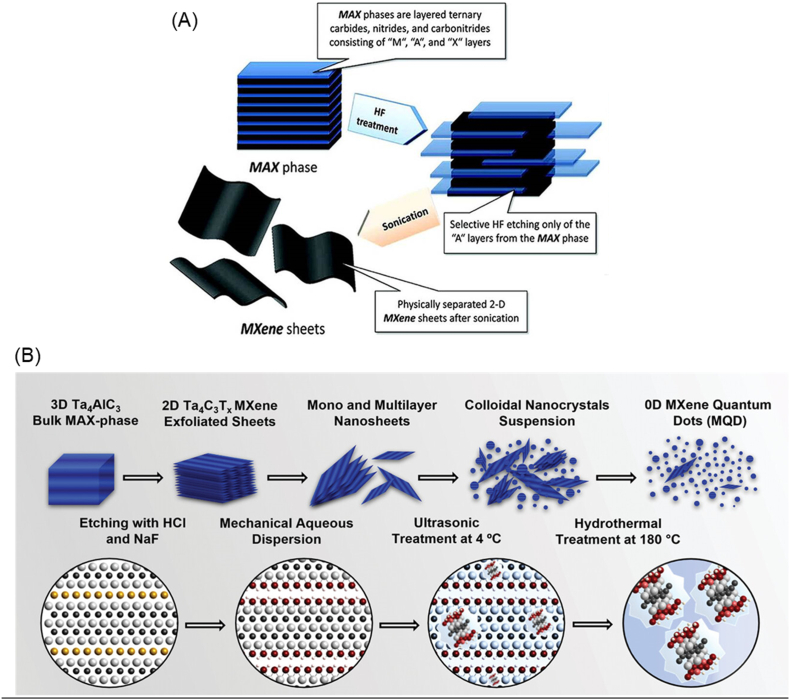
Schematic representation of the synthesis of MXenes from MAX phase by Top-down approach using (A) HF etching. This figure is reproduced with permission from Ref. [81] Copyright © 2012, American Chemical Society. (B) Fluoride salt-based etching of Ta_4_AlC_3_ MAX using HCl/NaF to synthesize Ta_4_C_3_T_x_ MXenes. Etching at 60 °C for 48 h resulted in the formation of exfoliated nanosheets, which were then subjected to ultrasonic sonication to obtain flakes ranging from multi-layered to mono-layered structures. These flakes subsequently underwent hydrothermal process to form Ta_4_C_3_ MXene quantum dots. This figure is reproduced with permission from Ref. [37] © 2021 The Authors. Advanced Functional Materials published by Wiley‐VCH GmbH.

For instance, exfoliation of tantalum aluminum carbide (Ta_4_AlC_3_) MAX phase is achieved through selective etching hydrochloric acid (HCl) and sodium fluoride (NaF) (Fig. 2B). When MAX is subjected to etching at 60 °C with HCl + NaF as etchant, the A-layer from the MAX is removed. This results in the formation of aluminum chloride (AlCl_3_) and sodium hexafluoro aluminate (Na_3_AlF_6_), which completes the exfoliation process (equation 1). In consequence, multilayered tantalum carbide (Ta_4_C_3_T_x_) MXene nanosheets are formed. The presence of NaF in the etchant generated abundant hydroxyl (-OH) and fluoride (-F) surface terminations, as illustrated in the proposed reaction chemistry (equations 1-3). Subsequent mechanical delamination methods of sonication and probe homogenization, cause partial oxidation of the reaction intermediates in the colloidal suspensions. Finally, the hydrothermal process (equations 4-6) yields quantum sized MXene dots and results in formation of tantalum oxide (TaO_2_ and Ta_2_O_5_). This series of chemical reactions, from etching to hydrothermal process imparts -OH and = O groups along with existing Cl-, F- and Na-based compounds as MXene surface terminations. During synthesis, the affinity of the MXene structure to oxygen and hydroxyl species from water and acids in the etchant, leads to -OH and = O groups on its surface. These functional groups, particularly -OH and -O, enhances the hydrophilicity of MXenes, resulting in increased wettability. The fluoride ions from NaF intercalate into the MXene structure, facilitating the cleavage of the M-A bonds exposing the surface to -OH and -F terminations. The increased presence of -OH groups stabilize the MXene structure, reducing oxidative degradation during long term storage. Thus, optimization of synthesis process allowed for size tailoring and surface terminations, enhancing the physiochemical properties of resulting materials. In the upcoming sections, we will discuss on how these surface terminations impart unique physiochemical properties and provide opportunities for further surface modifications to enhance their characteristics and applications.

1 2 Ta_4_AlC_3_ +6HCl +6 NaF → 2 Ta_4_C_3_ + Na_3_AlF_6_ + 3NaCl + AlCl_3_ + 3H_2_
2 2 Ta_4_C_3_ + 4H_2_O → 2 Ta_4_C_3_(OH)_2_ + H_2_
3 Ta_4_C_3_ +2NaF +2HCl → Ta_4_C_3_F_2_ +2NaCl + H_2_
4 Ta_4_C_3_ + O_2_ → Ta_4_C_3_(O)_2_
5 Ta_4_C_3_T_x_ + 7O_2_ → 4TaO_2_ +3CO_2_
6 Ta_4_C_3_T_x_ + 12OH- → 12 Ta_4_C_3_(OH)_2_ + 2Ta_2_O_5_

To gain insight into the physiochemical properties of MXenes, a comprehensive evaluation of their structure is imperative. MXenes are densely layered structures of micrometer size that, after removing their "A" layers, possess an accordion-like multilayer nanostructure. It is evident that M atoms form closely packed structures, and the X atoms occupy octahedral interstitial sites analogous to their parent MAX phases [11]. In addition, the structure of MXenes can be explained by differences in the number of M layers separating the A layers. For instance, there are two layers separating the A layers in M_2_AX, three layers in M_3_AX_2_, four layers in M_4_AX_3_ and so on [12] (Fig. 1B). This structural arrangement of the MAX phases gives them strong elastic stiffness, chemical resistance, good thermal and electrical conductivity with moderate coefficients of thermal expansion. Furthermore, the unique material properties of MAX phases are influenced by the combination of metallic-covalent M-X bonds and the relatively weak M-A bonds [13].

Etching is one of the important steps in the synthesis process which majorly affects the structural and chemical reactivity of MXenes. During this process, the weak M-A bonds are chemically etched from the MAX phase and the resultant hydrogen bonding and van der Waals forces formed after that impart stability to the synthesized MXenes with surface terminations [15]. As a result of relatively low number of atomic layers after the etching process, the thickness of a MXene stack is typically not more than 1 nm, whereas the lateral dimensions are between the nanometers to micrometers range, based on their synthesis [16,17]. The combination of interlayer spacing and surface terminations gives MXenes their distinctive qualities as a 2D nanomaterial, contributing to their remarkable electrical, optical, and magnetic properties [18]. Additionally, the inherent physiochemical properties of MXenes can be enhanced and customized by incorporating various compounds of interest through different modification techniques including spin, spray, dip, paint coating, painting and printing [[19], [20], [21]]. In comparison to other 2D materials, the modified MXenes have superior properties that allows them to play a prominent role in energy storage applications [22], water purification [23,24], chemical sensing applications related to heat transfer [[25], [26], [27]], photo/electrocatalysis [28,29], and electromagnetic interference shielding (EMI) [30]. Specifically, due to their versatile physicochemical properties, MXenes have significant potential to be employed in biomedical applications such as bioimaging [31,32], antibacterial [33], biosensing [34], drug delivery [35], immunology [[36], [37], [38]], tissue engineering [39], and other therapeutic methods.

In this review, the critical and most important aspects of the synthesis process of MXenes will be discussed. Further, this is the first review report that compiles the applications of MXenes for repair, regeneration and function of electroactive tissues and organs, including heart, nervous system, skin, bone and skeletal muscle.

## 2 Synthesis of MXenes

MXenes exhibits a diverse range of chemical compositions and structural arrangements [40] distinguishing them from other conventional 2D nanomaterials such as graphene [[41], [42], [43]], silicene [44,45], borophenes [46,47] and black phosphorene [48,49]. This structural and compositional versatility enables MXenes to possess a unique combination of properties, including exceptional electrical conductivity, mechanical flexibility, high surface area with substantial redox activity, and a narrow band gap. These characteristics set MXenes apart from other materials and make them highly promising for a wide range of applications in energy storage, electronics, and biomedical engineering [[50], [51], [52]]. The synthesis process of MXenes has a dominating role on the chemistry, layer structures, and surface functionalization of MXenes. This hereby creates the need to fully exploit the unique properties of MXenes. To date, different methods have been developed for synthesizing MXenes and their derivatives in a controlled manner. At present, the two main approaches which are being used to prepare MXene nanomaterials are: top-down approach and bottom-up approach*.* In the top-down approach, two-dimensional nanomaterials are prepared by breaking the van der Waals bonds between bulky 3-dimensional layers by different methods including mechanical exfoliation [53] and liquid-phase exfoliation [54]*.* Though the mechanical exfoliation methods are easy to use, the yield rate is very limited with low purity [55]. On the other hand, ultrasound-assisted liquid-phase exfoliation creates microbubbles, thus impeding the van der Waals forces within the layers, resulting in their separation with improved purity and yield [56,57]. Both these processes fall under the top-down approach as it begins with the bulky MAX phase as the source, and creates small, single to multilayered materials by using exfoliation methods. The exfoliation processes can either be sonication assisted or lithium (Li^+^) intercalation exfoliation or the dry exfoliation known as scotch-tape method [58].

The Bottom-up approach employs chemical means to assemble small molecules into 2D structure. This assembly and growth of multi-layered MXenes is carried out through the process of chemical vapor deposition (CVD) [59], molecular beam epitaxy [60] and pulsed laser deposition [61,62] involving high temperature and pressure. This highly expensive method produces high-quality MXenes compared to top-down approaches [63]. This is due to the fact that CVD creates the opportunity to adjust the reaction parameters, such as the number of layers, in contrast to the mechanical and liquid phase exfoliation processes. Yet, the preparation process for MXene with chemical vapor deposition and the other bottom-up approaches needs to be further optimized in terms of cost and complexity, for overall improvement in the quality of prepared MXenes [64,65].

In this section, we will discuss both top down and bottom-up approaches to get a sense of the physiochemical properties that can be obtained following these processes.

### 2.1 Top-down approach

MXenes are synthesized through the top-down approach which is carried out by a two-step process of chemical etching followed by the process of delamination [54]. Among the layered carbide, nitride, and carbonitride precursors, MXenes are created by selectively etching specific atomic layers [66]. The initial step in the synthesis process is influenced by the type of etching agents used, which can be classified based on their chemical makeup. These etchants include acidic solutions containing fluoride ions, such as hydrofluoric acid (HF) [50], a combination of Lithium fluoride-Hydrochloric acid (LiF-HCl) [67], and Ammonium hydrogen difluoride (NH_4_HF_2_) [68]. Additionally, there are fluoride-containing salts like lithium fluoride (LiF), sodium fluoride (NaF), potassium fluoride (KF) [69,70], and ammonium fluoride (NH4F) that are commonly employed as etchants [71] (Fig. 2A and B). The etching process is initiated by cleaving the metallic bond between M and A of the MAX phase by chemical etchant, which exposes the layers of “A” to be interleaved with ions of the etchant and its bulk precursors [54]*.* One of the easiest methods and most commonly employed method for etching MXenes is by using HF. However, HF solution is highly corrosive making etching unsafe for the users. Also, it limits the biomedical applications of the end products synthesized by this method because of the toxicity of the product. Hence, there is a need for optimization and development of newer methods using milder and non-toxic etchants. For example, HCl + LiF etchants produce fluoride ions that can effectively remove A layers from the MAX phase in a slow, controlled, and safe manner. In one study, Ti_3_C_2_T_x_ was prepared by mild etching conditions using HCl + LiF to generate a large lateral sized MXene nanosheets with a limited number of vacancies in its structural arrangements [72]. Furthermore, we developed Ta_4_C_3_T_x_ MXene Quantum Dots (TaMQDs) using HCl + NaF as an etchant and these TaMQDs were highly biocompatible and safe for biomedical applications [37].

Several studies have reported the differences in the composition of MXenes and their physicochemical properties when etching is done by HF versus the fluoride salt method. Notably the fluoride salt etching methods when compared to the HF method enhanced the properties of MXenes in the following way; 1) larger flake size and reduced number of defects compared to the defects by HF etching; 2) the presence of cations (Li^+^, Na^+^, K^+^, and NH_4_^+^) in the etching solution increased the interplanar space by placing themselves between the interleaved layers and 3) augmented the quantity of oxygen functional groups on the MXenes surface [37,[72], [73], [74]]. As a result of which synthesis of MXenes using fluoride-based salt etchants is highly sought after to achieve superior electrical conductivity, large flake size, and enhanced mechanical properties of the end product*.* Consequently, it is essential to develop other etchants to replace HF in order to ensure the safe usage of MXenes in biological applications. Mild fluorine-based etchants, as well as fluorine-free methods, could also be employed to achieve this goal. Recently, in our lab, we developed fluorine-free Ta_4_C_3_T_x_ MXene hybrids by employing a modified alkaline-based etching system containing hydrochloric acid (HCl) and potassium hydroxide (KOH) for an acidic (HCl)/alkaline (KOH) treatment [75]. The Ta_4_C_3_T_x_ MXene hybrids demonstrated improved electrochemical performance and biocompatibility, highlighting their strong potential for use as bioelectrodes in supercapacitor systems. These advancements suggest they could be highly effective in energy storage applications.

After etching of MXenes, the next step in the synthesis process is to delaminate it into multilayered composite. Currently, two methods are available for delamination, and these are delamination assisted via mechanical methods (Fig. 2A) and delamination assisted via intercalation [72]. It has been reported that mono-layered or very few layered MXenes are hard to obtain using the first method, because multi-layered MXenes exhibit intense interlayer interactions, making the separation of such layers difficult [76]. As a result, single-layer MXene flakes are produced exclusively through intercalation, as the multi-layered structure of MXenes allows various ions and molecules to insert between the layers, making it difficult to separate them into individual layers*.* To further facilitate this process intercalated MXenes are sonicated in deaerated water to get single-layered MXene sheets [77,78]. Adding on to the above-mentioned concept of intercalation, HF-synthesized MXenes require organic compounds as intercalants in order for the multi-layers to be effectively separated [16,17]. They can be polar organic compounds [79] like isopropylamine, dimethyl sulfoxide (DMSO), or organic base molecules such as hydrazine, urea, and tetrabutylammonium hydroxide (TBAOH). Amongst these TBAOH, isopropylamine and DMSO are widely used as intercalating agents for Ti, V and Nb based MXenes [12,77,80]. It is also possible to get single layer MXenes by using etchants such as LiF + HCl or NH_4_F, where the NH^+^ and Li^+^ ions can be inserted between layers. This eliminates the need for the use of organic intercalating compounds to obtain single-layer MXenes. This results in MXenes having a longer intermembrane space between the stacks than those that are produced by HF, and the subsequent single-layer MXenes are easily achieved by sonication, and this removes the need for additional intercalation [72].

### 2.2 Bottom-up approach

MXenes can be created by controlling their composition and morphology at the atomic level through the bottom-up approach [82]. In this approach, CVD [63,83] and wet-chemical synthesis [84,85] are the most common methods employed to date to develop MXenes with high quality and purity. The CVD growth approach designed for transition metal carbides is both efficient and adaptable, enabling the synthesis of a wide range of 2D ultrathin transition metal carbides and nitrides with exceptional physiochemical characteristics. Some of the key advantages of bottom-up synthesis methods over top-down approach are that they allow the manipulation of size distribution, morphology and the surface termination of MXenes in a precise manner, which are crucial parameters to be considered when MXenes are used for biomedical applications. For instance, using CVD, Ren and colleagues reported the growth of ultrathin -Mo_2_C MXenes with fewer defects and high quality [86]. During this process, at temperatures above 1085 °C, thickness, surface coverage, and morphology of Mo₂C flakes was controlled by varying the Cu/Mo ratio, flow rates, and carrier gas types. Higher Cu ratios (0.5–2) improved coverage (19–68 %) and flake size (up to 30 μm), while adjusting gas flow rates and types enabled thickness control (7–150 nm) and optimized flake shapes for applications in supercapacitors and transistor contacts. Also, a similar CVD process was employed to obtain tungsten carbide and tantalum carbide (TaC) crystals by substituting W or Ta foils in place of Mo foils respectively [63]. Thus, CVD process can be used to yield versatile MXene crystals with superior physiochemical properties making them high-quality 2D nanomaterials. However, the MXenes prepared by the bottom-up approach are multi-layered, and mono-layered MXene stacks cannot be yielded using this method. This creates the need to develop better and more refined techniques to improve the bottom-up synthesis protocols.

### 2.3 Comparison of MXene synthesis with other 2D materials

As a promising class of 2D materials, MXenes offer several advantages in their synthesis processes compared to other 2D counterparts. For instance, unlike graphene [87], which requires high-temperature CVD or mechanical exfoliation methods with limited scalability and high production costs, MXenes are synthesized through a more straightforward and cost-effective top-down approach using elective etching [88]. This selective etching method contrasts with the less expensive liquid-phase exfoliation methods used for graphene and TMDs (transition metal dichalcogenides) [89]. TMDs synthesized by liquid-phase exfoliation methods have more defects, lower yields, higher contamination by chemical groups and potential phase transition of the TMDs after exfoliation. Furthermore, compared to graphene and TMDs, MXenes are held together by van der Waals bonding between the MXene interlayers, making them form stable nanostructures [9]. h-BN (hexagonal boron nitride) demands high energy and processing time for its synthesis by mechanical, chemical and liquid-phase exfoliation methods [90]. h-BN synthesized via mechanical exfoliation suffer low yield affecting its scalability [91]. Chemical and liquid-phase exfoliation methods employing gaseous and liquid precursors are prone to produce toxic byproducts [92] and hydrolyze readily during synthesis [93], respectively.

MXene synthesis, on the other hand, primarily employs top-down approach, where the potentially hazardous HF that is traditionally used, can be substituted for safer and more scalable methods using milder etchants like HCl and LiF. The surface terminations yielded from this chemical etching method also allows for easy surface modifications, providing unparalleled versatility for functionalization unlike other 2D materials. The presence of surface terminations and ease for surface modifications, contributes to MXenes’ unique physiochemical properties expanding their promising applications across various fields. Thus, we see that this combination of safer synthesis with enhanced properties and highly tunable surface distinguishes MXenes from other 2D materials.

## 3 Harnessing MXene properties for Innovations in tissue engineering

MXenes have emerged as exceptional candidates for advancing the field of tissue engineering and revolutionizing biomedical applications because they exhibit excellent hydrophilicity, thermal conductivity, chemical stability, electrical conductivity, and biocompatibility [94]. Furthermore, MXenes possess very good metallic properties, that, in turn contribute to their optical, electrical, and magnetic properties. Therefore, MXenes and MXene-based nanomaterials are becoming very popular in the field of bioelectronics and other biomedical applications. To design 2D nanomaterials for a specific function and application, their physiochemical properties are altered and tailored by surface functionalization. Therefore, in this report we will also discuss the importance of functionalization in enhancing the biomedical properties of MXenes.

### 3.1 Biocompatibility

MXenes are chemically composed of relatively safe elements such as carbon, nitride, and a few early transition metals, which are inert in nature to the living organisms and this makes MXenes safe for use in biological systems [95,96]. Several reports in rodents suggest that MXenes can be made biodegradable after *in vivo* administration and can be excreted from the body without causing any complications [16]. It is important to note that MXenes by virtue of their composition possess excellent hydrophilicity and biocompatibility, which in turn are related to each other. In fact, the hydrophilicity of a biomaterial enhances its biocompatibility, making it ideal for therapeutic applications. With respect to biomedical applications, several reports suggest that MXenes as highly safe for *in vitro* [95,[97], [98], [99], [100]] and *in vivo* [17,32,101,102] applications to the biological systems. Due to excellent biocompatibility MXenes have created a new gateway for applications as biosensors for biological devices [103,104]. MXenes, are also being used to fabricate wearable gas sensors, which operate efficiently at room temperature without causing any toxicity [26,27]. Furthermore, MXenes are used as coatings for strengthening the implants for long term use, owing to their inert nature and immuno-compatible nature [39,105,106].

However, to further improve the biocompatibility of MXenes, its surface can be modified with various functional groups. In this regard, the surface functionalization of nanomaterials with alkoxysilanes is widely reported, due to their inherent ability to form covalent bonds with organic and inorganic materials [98,[107], [108], [109]]. The silane coupling agents contain a variety of functional groups, which include amino, methacrylate, epoxy, vinyl, chloro, and phenyl. These groups facilitate the interaction of MXenes with hydrophilic and hydrophobic polymers and form nontoxic composites [110]. Another modification of MXenes, which significantly enhances their biocompatibility, is by incorporation of natural and synthetic polymers. Chitosan is an efficient natural polymer that has been widely used for biomedical applications due to its biodegradability and biocompatibility [[111], [112], [113]]. Chitosan has been shown to significantly enhance the biocompatibility of MXenes when conjugated with it [114]. A recent report from our laboratory demonstrated that a conjugation of chitosan and MXene promoted survival of cells when grown on this composite. This 3D heterostructure containing chitosan and MXene is highly desirable platform for cell delivery and future applications in cell therapy mediated tissue repair [115]. Furthermore, functionalization of MXenes with several synthetic polymers such as soybean phospholipid, Polyvinyl alcohol (PVA), and polyethylene glycol (PEG) enhanced the biocompatibility and physicochemical stability of MXenes [116,117]. Additionally, the application of different coatings can not only enhance biocompatibility but also reduce MXene oxidation during long-term *in vivo* applications, while serving as a reliable platform for drug delivery. Recently, the concept of MXene-polydopamine-antibody (MXene-PDA-Ab) formation was developed [118]. This approach demonstrated not only high biocompatibility but also a stable state in aqueous solutions, along with high affinity and specificity towards targeted cancer cells. Given the non-selective nature of PDA coating and monoclonal antibody (mAb) binding, this process can be employed to modify various types of MXenes. This adaptability paves the way for novel diagnostic and therapeutic strategies.

### 3.2 Optical properties of MXenes

MXenes possess strong plasmonic resonances, wide optical transparency windows, non-linear optical performance, photothermal conversion and adaptive optical responses, this contributes to their excellent optical properties [119,120]. The surface terminations on MXenes affect their electronic structure which in turn has a vital role in altering the optical properties [121]. Hence It is essential to understand the importance of MXenes’ surface functional groups in influencing the absorption, emission, scattering, saturation, absorption, non-linear refractive index, transmission and photoluminescence. The optical characteristics of MXenes are heavily influenced by their energy structure, which includes factors such as the energy band gap, direct and indirect band gaps, as well as their topological properties [122,123]. A fundamental optical property of MXenes is the energy band structure, which is heavily influenced by the surface groups, presence of external electrical field, stress, and doping of other optically active materials [40]. Interestingly, different MXene structures have different optical properties influencing their application of these materials in the field of biology. According to Xue et al., MQDs possess better photoluminescence (PL) properties compared to 2D MXene nanosheets, as they have fewer defects and higher redox capacitance, which makes them a better photoactive material with a longer circulation time *in vivo* [124]. Notably, the TaMQDs developed in our lab exhibited excellent optical properties and thermal stability [67]. We demonstrated that the enhanced surface properties and ideal size of these MQDs facilitated their uptake by endothelial cells to exhibit their immunomodulatory role in preventing allograft vasculopathy *in vivo*. Furthermore, the doping of photoactive materials enhances the optical and physicochemical properties of pristine MXenes. In this regard we recently reported that GerMXene heterostructure, that was developed in our lab by conjugating MXene nanosheets with germanane quantum dots, possess superior optical properties. The addition of germanane quantum dots not only enhanced the optical properties of the final product, it also improved the surface area, stability, and dispersibility of GerMXene in aqueous solutions [115].

The large surface area of MXenes allows them to have a wide wavelength range, spanning from UV to NIR, with a high efficiency of conversion of light to heat for anti-cancer photothermal therapies. Additionally, the ability of NIR light to penetrate deep into the target cancer tissues results in a rapid and effective treatment with minimal invasiveness. These advantages have further highlighted the potential of MXenes as a major biomedical tool in the field of cancer theranostics [16,17]. To generate effective tools, improvements in intrinsic optical properties can be achieved by surface functionalization through self-initiated photopolymerization process by coating polymers and self-assembled monolayers on the surface of MXenes [101,[125], [126], [127]]. Also, the optical properties of MXenes can be improved significantly by adjusting the interlayer distance through intercalation [128]. Both ion-mediated intercalation [129] and molecule-mediated intercalation (e.g. TMAOH) [130] can tailor optical properties of MXenes to fit them into the UV light range from visible light absorption spectra [131,132]. Overall, excellent optical properties of MXenes offer potential benefits across a range of biomedical applications, including but not limited to photocatalysis [133], sensing [134], photoacoustic imaging [117], photothermal therapy [135], photodynamic therapy [116] and applications related to controlled drug release [35].

### 3.3 Electrical properties of MXenes

MXenes show excellent conductivity in comparison to many other 2D nanomaterials [100,136]. As mentioned earlier, the physio-chemical properties of MXenes can be significantly influenced and tailored as per the application, by its fabrication process. With respect to conductivity of MXenes, the fabrication and delamination processes facilitate conductive nature of MXenes and this in turn impacts its topological insulative nature [81,137]. Interestingly, in bare monolayers, MXene molecules have transition metals that greatly improve their electrical conductivity, and the surface terminations (OH/O/F) facilitate a metal-semiconductor transition making it either a conductor or a semiconductor. In addition, for the optimal performance of MXenes in any electronic application, the method of preparation, the type and nature of surface termination [138], the internal structure, the elemental composition [139], as well as environmental conditions such as humidity, pH and temperature [140] should be carefully evaluated, in order to obtain maximum benefits from its physicochemical properties. For instance, functionalization of metal ions with MXenes improves electron-donor surface groups, thereby improving their electrical conductivity [141]. Additionally, adjusting the oxidation state of the transition metal in Ti_3_C_2_T_x_, such as by doping with -NH_2_ [142], -NO_2_ [143], and -SO_3_ [144], the electrical property of MXenes can be enhanced. In a study by Lu et al., they found that the treatment of MXene with urea resulted in N functional substituted MXene (HND), and this facilitated better oxidation state and larger interlayer spacing of MXene [145]. This property allowed higher capacitance, improved ion transport and enhanced charging rate of HND compared with the pristine Ti_3_C_2_T_x,_ stating the significance of N_2_ doping in enhancing the electronic properties of MXene composites.

Another potential way to further enhance electrical properties of MXenes is by coating them with conductive polymers, which will intercalate between its layers, increasing the spacing between them for efficient electron transport. In this regard, a conductive polymer, polypyrrole (PPy), has been used by Lu et al. to intercalate Ti_3_C_2_T_x_ layers to obtain remarkable gravimetric/volumetric, high energy power densities and demonstrated excellent electrical properties. Several other studies have been carried out where MXene surfaces have been polymerized with other conductive polymers, such as Polyaniline (PANI) [142,146], Polyacrylamide (PAM) [147,148] and PPy [[149], [150], [151]]. These studies highlighted the possibilities and importance of functionalization of MXenes to further improve their interlayer spacing and electroconductive performance. Due to the excellent electrical properties MXenes are being used in bioelectronics. Some of the examples of this are the development of wearable electronic devices for monitoring and controlling physiological signals in the body [[152], [153], [154]]. The electrical conductivity of MXenes also facilitates its application as biosensors to measure analytes of interest in a suspension or biological environment [155,156]. Additionally, MXenes can also be used as filler materials in non-conductive scaffolds to prepare electroconductive constructs that can be employed for the repair and regeneration of electroactive or electrosensitive tissues [8]. T.N. Koltunowicz et al. investigated the capacitance, inductance, and phase shift angle studies of the MXene-PCL nanocomposite with the aim of understanding the mechanism of electron transport and conductivity [157]. They demonstrated that the frequency dependence of the capacitance exhibited a clear, sharp minimum in the frequency range of 50 Hz to over 10^4^ Hz, while the frequency dependence of the inductance showed sharp maxima, the position of which exactly coincided with the position of the minima for the capacitance, indicating the occurrence of parallel resonances. Current conduction occurs by electron tunneling between nanoparticles. In the frequency range from approximately 10^4^ Hz–10^5^ Hz, the inductance exhibited a broad minimum. The position of this minimum coincides exactly with the position of the maximum of the phase shift angle – its amplitude is close to 90°. It was found that the average value of the distance over which the electron tunnels was determined with some approximation to be about 5.7 nm and the expected value of the relaxation time to be *τ*_*M*_ ≈ 3 × 10^−5^ s. Further elucidations are discussed in detail in the upcoming sections.

### 3.4 Magnetic properties of MXenes

Magnetic properties of MXenes have been studied less extensively compared to other features, such as electrical and optical properties. Based on the research conducted to date, MXenes exhibit a range of magnetic properties. Interestingly, different types of MXenes have shown different magnetic properties such as Ti_2_C, Ti_2_N [158], and Cr_2_C [159] are reported to have ferromagnetic properties [160], while Ti_3_C_2_ and Cr_2_TiC_2_F_2_ possess anti-ferromagnetic properties [8,161,162]. The magnetic properties of MXenes, like their optical and electrical characteristics, are influenced by several factors. One key factor is the composition of the "M" element in the original MAX phase, which plays a significant role in determining their magnetic behavior [163], the interlayer thickness [164], surface termination, and surface functionalization [117,165]. Apart from these, the magnetic properties of MXenes can also be influenced by their external conditions. Applying an external electric field to a Ti_2_C monolayer results in a transition from an antiferromagnetic semiconductor state to a range of other magnetic states, including ferromagnetic semiconductor, half-metal, magnetic metal, non-magnetic metal, or non-magnetic semiconductor [166].

The magnetic properties of MXenes are especially useful in the field of electromagnetic interface (EMI) shielding. MXenes are capable of showing a number of interesting properties such as increased electromagnetic radiation absorption efficiency and a sufficiently high level of surface reflection [30,167,168]. However, some of the MXenes in their pristine state are non-magnetic in nature, primarily due to their high conductivity. Hence, this non-magnetic nature limits their application in EMI shielding. In order to overcome this, MXenes are conjugated with polymers to increase their magnetic properties [150,162,[169], [170], [171]]. Apart from being used as EMI shielding materials for biomedical research, the magnetic properties of MXenes are employed to fabricate excellent contrast agents for bioimaging applications [124,172,173]. Therefore, MXenes have the potential to be used for next-generation technologies for nanomedicine, however, further research is required to find the best suited MXenes for any specific application.

### 3.5 Photothermal properties of MXenes

In the recent years, MXenes have gained widespread attention for their notable ability to convert light-to-heat, finding applications across various energy-related [174] and biomedical fields [175]. This property stems from MXenes’ strong light absorption in the near infrared (NIR) region, making them particularly beneficial in photothermal therapy (PTT). In case of energy-based applications, MXenes are effective in converting sunlight into heat for photothermal water evaporation [176] and actuator systems [177]. In biomedical applications, MXenes demonstrate significant potential in localizing heat to target cancer cells, causing tumor ablation while minimizing damage to healthy tissues. MXenes demonstrate usefulness in cancer management in the form of nanosheets and quantum dots [117,178,179]. They serve as both efficient photothermal agents (PTA) to modulate tumor progression and contrast agents to enable photoacoustic (PA) imaging. This dual functionality enhances visualization during cancer diagnosis, effectively bridging the gap between diagnostic imaging and therapeutic interventions.

Surface modified MXenes are found to exhibit enhanced PT ability. By integrating metal oxides and nanoparticles, like manganese oxide (MnO_x_) [180], gold (Au) [181] and iron (Fe) [182], Ti_3_C_2_T_x_ MXenes served as multimodal magnetic resonance imaging (MRI) and PA for enhanced PTT. Further, platinum (Pt) nanoparticles loaded PEGylated MXenes were found to induce apoptosis and necrosis, while enhancing PT effect. This aided in effective synergistic tumour ablation via PTT and nanocatalysis, contributing to improved photothernaostics [183]. MXenes have also been used as drug delivery agents [184,185] for targeted tumor therapy, leveraging the combined effects of PTT and targeted drug administration. The energy released by MXenes during PTT facilitates drug release and enhanced deeper penetration into tumors, leading to improved tumor eradication. Collectively, these photothermal attributes of MXenes position them at the forefront of cancer theranostics, promising future avenues in translational research.

### 3.6 Antibacterial properties of MXenes

MXenes’ antibacterial properties was first investigated in 2016, concentrating on their activity against different bacterial strains [33]. The unique physiochemical properties of MXenes enable them to cross biological barriers, including bacterial cell wall. This breach damages bacterial cell leading to cytoplasmic content release, which produces reactive oxygen species (ROS) and causes oxidative stress [186]. Compared to graphene and graphene oxide, MXenes sustain augmented antibacterial properties, efficiently inhibiting bacterial growth at an accelerated rate in both time-dependent and dose-dependent fashion [187]. Additionally, polymer-conjugated [188] and metal oxide- [189] integrated MXenes have been experimented for antibacterial effects, with these modifications attributed to their enhanced antibacterial properties against both Gram-positive and Gram-negative strains.

Most recently, it has been observed that the photothermal and photoacoustic properties of MXenes [190] can be exploited to improve their antibacterial properties. This capability is applied in wound healing applications to develop wound dressings with antibacterial properties through conjugation with hydrogels [191]. Electrospun chitosan nanofibers with MXenes were used to construct natural polymeric bandages, achieving an antibacterial activity of approximately 95 % against *Escherichia coli* and 62 % against *Staphylococcus aureus* [188]. Furthermore, polyvinyl alcohol (PVA)/MXene hydrogel was utilized for wound repair to prevent infection at the injured site. This composite hydrogel system demonstrated excellent water retention, accelerating wound healing, while leveraging its hyperthermic mechanism to achieve bacterial inhibition rate of over 98 % against *E. coli* and 95 % against *S. aureus* when exposed to NIR irradiation [192]. Similarly, polylactic acid (PLA) modified MXenes, when subjected NIR irradiation, directed a bacterial inhibition rate of 99.9 % [193]. This effect is attributed to the photothermal conversion ability of the MXene system, which raised the temperature to 50 °C, resulting in damage to the bacterial cell wall. These studies indicate that MXenes pave the way for innovative antibacterial solutions for wound repair and regenerative applications.

## 4 Applications of MXenes to repair electroactive tissues and organs

As discussed in the above sections MXenes possess several unique properties because of their chemical composition and structural arrangement. As a result, MXenes have been used for a variety of biomedical applications [[213], [214], [215], [216]]. A comprehensive overview of these distinctive physiochemical properties arising from their surface terminations and modifications, along with their application in different biomedical fields, is summarized in Table 1. The exceptional conductivity and excellent electrical, optical and electromagnetic properties of MXenes, has positioned them at the forefront of bioelectronics. Additionally, MXenes' potential for repairing electroactive tissues such as brain, heart, skin, bone and skeletal muscle, has gained significant attention. In this section, we have reviewed the advancements in the application of MXenes for the repair of electroactive tissues in the form of composite scaffolds and in bioelectronics. Also, at the end of this section we have presented ‘Table 2’ and ‘Table 3’ that list some of the current MXenes and their derivatives employed in bioelectronics and tissue engineering applications.

**Table 1:** Comprehensive overview of MXenes - surface modifications, properties, and applications.

MXene	Surface Modification	Physicochemical Properties	Biomedical Applications	References
Ti_3_C_2_	Hydroxyl (-OH), Oxygen (-O), Fluorine (-F)	• High electrical conductivity • Hydrophilicity • Flexibility • Photothermal capabilities	Neural tissue engineering, Antibacterial and Photothermal applications	[[194], [195], [196], [197], [198]]

Nb_2_C	Bioglass ceramic incorporation	• Osteoconductivity, • Osteoinductivity, • Tumor ablation properties	Bone regeneration, Tumor growth inhibition	[199]

Ta_4_C_3_	Fluorine-free etching	• Enhanced electrical properties • Biocompatibility	Bioelectronics, supercapacitors	[37,75]

Ta_4_C_3_/IONP/SP	Incorporation of Iron oxide nanoparticles (IONP) and modified with soybean phospholipid (SP)	• Enhanced multimodal imaging for magnetic resonance imaging (MRI) and X-ray computed tomography (CT). • Increased stability under physiological conditions	Cancer theranostics	[117]

Ti_3_C_2_-PEG	PEGylation	• Biocompatibility • Promotion of synchronized cardiomyocyte beating and gene expression	Cardiac patches for myocardial infarction	[200]

Ti_3_C_2_-Chitosan	Conjugation with natural polymers	• Enhanced biocompatibility • Biodegradability • Structural stability • Antibacterial property	Cell delivery, Wearable biosensors, and Tissue engineering	[114,188,201]

Ti_3_C_2_-gold nanoparticles	MXene-based biosensor with immunosensor sandwiched with MXenes and gold nanoparticles	• Immuno-sensing of biological analytes even at low concentration	Biosensor for cardiac biomarkers	[202]

Ti_3_C_2_-Ab	Functionalization with antibodies	• High biocompatibility • Stability in aqueous solutions, • Specific biomarker detection	Cardiovascular and Bone regeneration biosensors	[203,204]

Ti_3_C_2_-PLA	Combination with polylactic acid (PLA)	• Improved mechanical rigidity, • Enhanced hydrophilicity • Promotion of osteogenic differentiation • Antibacterial property	Scaffolds for Bone regeneration and Wound repair	[98,193,205]

Ti_3_C_2_-PCL	Integration with polycaprolactone (PCL)	• Increased hydrophilicity • Increased Conductivity • Enhanced protein adsorption and cell attachment	Cardiac and Bone tissue engineering	[206]

Ti_3_C_2_-PANI	Conductive polymer intercalation	• Enhanced electro-conductivity • Enhanced interlayer spacing	Bioelectronics, Biosensors	[207,208]

GerMXene	Doping of Germanane quantum dots	• Improved optical properties • Enhanced surface area • Stability in aqueous solutions	Bioimaging, Optical biosensors	[115]

MXene-PVA	Incorporation of polyvinyl alcohol (PVA)	• Biocompatibility • Photothermal/Photoacoustic/Chemo therapeutics • Antibacterial property	Wound repair, Cancer therapy	[116,192]

MXene-PLLA	MXene incorporate with poly-L-lactic acid (PLLA)	• Hydrophilicity • Surface roughness • Superior conductivity • Synchronous electrical stimulation	Neural Tissue engineering	[209]

MXene- UHAPNWs	Incorporation of Hydroxyapatite ultralong nanowires	• Osteo-inductivity, • Osteo-conductivity	Bone regeneration in calvarial defects	[210]

MXene/GelMA	Gelatin methacrylate (GelMA) hydrogel formulation	• Promotes photothermal tumor ablation • Promotion of osteogenesis • Antibacterial property	Post-surgical tumor and Bone lesion repair	[211]

MXene/HA NF	Hydroxyapatite (HA) nanoparticles in electrospun nanofibers (NF)	• Enhanced photothermal property • Controlled ion release	Bone repair and osteogenesis under NIR irradiation	[212]

MXene/Metal oxides and metal nanoparticles	Integration of oxides like Manganese oxide, gold and iron nanoparticles	• Enhanced Photothermal property • Photoacoustic property	Cancer theranostics	[[180], [181], [182]]

**Table 2:** MXenes and their derivatives as emerging materials in diagnosis of electroactive tissues.

MXene composite	Area of application	*In vitro/in vivo* models	Highlights of the study	Reference
Ti_3_C_2_T_x_/Pt nanoparticles/glass carbon electrode (GCE)	Neurodegenerative disorders	phosphate buffered saline (PBS)/human urine	The detection limits of Dopamine containing uric acid and ascorbic acid are 0.48 and 0.38 μM, respectively	[266]

Ti_3_C_2_T_x_ sheets/Co_3_O_4_ hexagons	Neurodegenerative disorders	PBS/human urine	Wide detection range of isoprenaline ranging from 0.01 to 0.33 μM High sensitivity towards isoprenaline in harsh environment	[267]

Au nanoparticles/Ti_3_C_2_T_x_ nanosheets@poly(amidoamine) dendrimers	AMI	human serum samples	Wide range of cTnT detection even after 3 weeks of storage	[268]

Ti_3_C_2_T_x_/glucose oxidase/poly(3,4-ethylenedioxythiophene) (PEDOT):4-sulfocalix [4]arene	Heart disease	PBS & Commercial fruit juice	Linear range detection of glucose from 0.5 to 8 mM	[269]

Horseradish peroxidase/Ti_3_C_2_/Nafion modified GCE	AMI	PBS/human serum	Wide detection range of H2O2 from 5 to 8000 μM with a low detection limit of 1 μM in clinical serum samples	[270]

Chitosan/cholesterol oxidase/Ti_3_C_2_T	CVD	Human serum	Linear detection range of cholesterol ranging from 0.3 to 4.5 nM with a low detection limit of 0.11 nM Also, it was highly sensitive towards cholesterol with a value of 132.66 μA nM^−1^ cm^−2^	[271]

Ti_3_C_2_T_X_ – MWCNTs/Valinomycin/PET/Ag	High blood pressure and strokes	Human skin	Displayed a large surface area with excellent charge transfer rate High sensitivity of the sensor for [K^+^] ion in the range of 63–173 mV/dec	[272]

Ti_3_C_2_T_X_/PEDOT: PSS films/sodium ion selective membrane/Ag/Au	Heart failure	Human Skin	A shelf life of 50 days with good electrical performance of upto 1000 cycles. It exhibited 40 mV of sensitivity towards [Na^+^] ion	[273]

Ti_3_C_2_T_x_/laser-burned graphene/Polydimethylsiloxane/cortisol antibody	Cardiovascular & Skeletal muscle disorder	Human sweat	Exhibited linear detection range of 0.01–100 nM with a low detection limit of 88 pM for cortisol	[274]

Au nanoparticles/Ti_3_C_2_T_x_/GCE/Alkaline phosphatase (ALP) Antibody	Skeletal growth and bone mineralization	Human serum	The dynamic range is wide, ranging from 10.0 to 1500 U/L, with a detection limit of 4.0 U/L for ALP	[204]

fluoroalkyl silane functionalized Ti_3_C_2_T_x_/polyaniline/Ag/polyethylene terephthalate (PET)	Monitoring the pH of human sweat	Human skin	An improved real-time monitoring system to measure the pH value of sweat during exercise that is responsive, sensitive, and reversible	[275]

**Table 3:** MXenes and their derivatives as emerging materials in regeneration of electroactive tissues.

MXene composite	Area of application	*In vitro/in vivo* models	Highlights of the study	Reference
Ti_3_C_2_T_x_ MXene nanosheets	Neural tissue engineering	Mouse neural stem cells	Neurite outgrowth and Ca^2+^-gated channels increased In addition, they have mature properties, including enhanced synaptic transmission	[276]

2D V_2_C MXenzyme	Neurodegenerative disorders	mice fibroblast cell line (L929 cells) & neuronal cell line (PC12 cells)/mice ear & ankle inflammation model	Protects against oxidative stress *in vitro* when combined with superoxide dismutase, catalase, peroxidase, glutathione peroxidase, and thiol peroxidase Relives ROS stress induced damage *in vivo*	[277]

GelMA/Ti_3_C_2_T_x_ hydrogel	skeletal muscle tissue engineering	murine myoblast cell line (C2C12)	Adding MXene to GelMA hydrogel improved its conductivity and rheological properties In comparison to GelMA only hydrogels, C2C12 myoblast differentiation was enhanced	[278]

Ti_3_C_2_T_x_	skeletal muscle tissue engineering	Mouse macrophage cell line (RAW 264.7), C2C12, human umbilical vein endothelial cells (HUVECs)/SD rats	In vitro, MXenes have demonstrated anti-inflammatory properties, improved differentiation of myoblasts, and promoted angiogenesis MXene transplantation *in vivo* helped in skeletal muscle regeneration in a full-thickness muscle defect model	[279]

GelMA/β-TCP/sodium alginate (Sr^2+^)/Ti_3_C_2_	osteomyelitis infection	rat bone marrow mesenchymal stem cells (rBMSCs)/SD rats	Photothermal induced antibacterial activity with excellent biocompatibility Facilitated bone regeneration both *in vitro* & *in vivo*	[280]

aromatic thermosetting copolyester/Ti_3_C_2_T_x_	Osteoarthritis	Rat MSCs	The composite exhibited excellent tribological properties, adhesiveness and hardness for bone and joint application The composite was biocompatible towards MSCs	[281]

poly(L-lactide-co-ε-caprolactone)/col/Ti_3_C_2_T_x_	Bone tissue engineering	Mouse preosteoblastic cell line (MC3T3-E1)	The nanofibrous scaffold improved the survival, growth and osteoblastic differentiation of MC3T3-E1 compared to control	[282]

Ti_3_C_2_T_x_	Bone tissue engineering	MC3T3E1/SD rats	MXene enhances the osteoblastic differentiation compared to control *in vitro* MXene helps in osteo-induction and bone regeneration of the bone defect *in vivo*	[283]

Ti_3_C_2_T_x_	Bone tissue engineering	Human MSCs	MXenes promoted the osteoblastic differentiation of MSCs in concentration less than 20 μg/ml	[284]

Oxidized alginate/gelatin/Ti_3_C_2_T_x_	Skin tissue engineering	mouse fibroblast (NIH3T3s)	The hydrogel facilitated attachment and growth of NIH3T3s cells for a period of 7 days	[285]

### 4.1 Application of MXenes in neural tissue engineering

#### 4.1.1 MXenes in bioelectronics for neural tissues

**Fig. 3:**
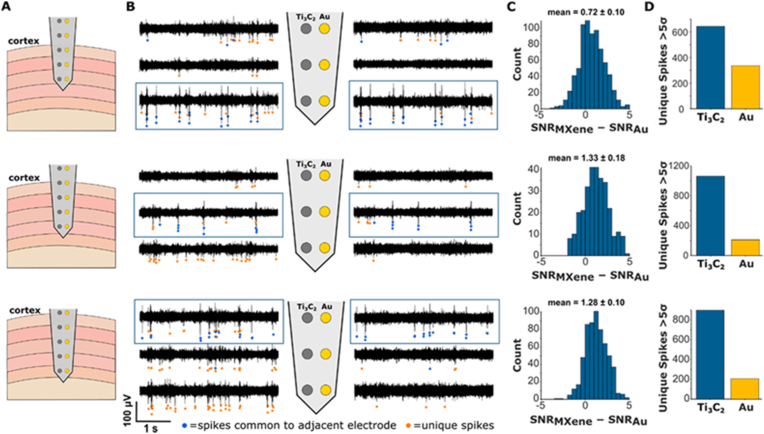
Comparison of *in vivo* signal recorded intracortically with Ti_3_C_2_ MXene microelectrode and Au electrodes. (A) Schematics of an intracortical electrode array with five Ti_3_C_2_–Au stereotrode pairs. The array was inserted into the cortex and advanced in approximately 500 μm steps to a depth of 2 mm while recording. (B) Multi-unit spiking activity observed on the distal three pairs of electrodes in the array at three different recording depths. Spikes that were observed simultaneously on adjacent Ti_3_C_2_ and Au contacts are indicated by blue dots. Spikes unique to a given electrode are indicated by orange dots. (C) Histogram of SNRMXene–SNRAu for the common spikes on the adjacent electrodes outlined in blue boxes in panel B. In all cases, the distribution mean was significantly greater than zero (permutation tests, p < 0.001), indicating higher SNR on Ti_3_C_2_ contacts compared to Au. (D) Number of unique spikes seen on adjacent Ti_3_C_2_ and Au electrodes. In all cases, more unique spikes were observed on Ti_3_C_2_ contacts, indicating that these electrodes were more sensitive for recording unit activity than Au electrodes. This figure is reproduced with permission from Ref. [196]. Copyright © 2018, American Chemical Society.

In the last few years, advancements in materials and electronics science have spawned a new generation of bioelectronics for medical theranostics, health monitoring devices and wearable devices. In this regard, the exceptional physical, chemical, and electrical properties of MXenes have created considerable amount of interest in this field. Compared to other known carbon-based nanomaterials such as graphene and its derivatives, MXenes exhibit a high volumetric capacitance and electrical conductivity, making it a promising material for neural interface applications. In this regard, Xu et al. devised a highly sensitive Ti_3_C_2_-MXene field-effect transistor (FET) for detecting neurotransmitters (dopamine) and to measure action potentials from hippocampal neurons [195]. This high-performance, highly biocompatible MXene biosensor enabled instantaneous, label-free monitoring of neuronal spikes, which detected dopamine at extremely low levels (100 × 10^−9^ M) compared to other graphene-based sensors. Similarly, in a study by Driscoll et al. [196], the performance of Ti_3_C_2_ MXene microelectrode was compared with gold microelectrodes for neural sensing and recordings as shown in Fig. 3. In this study, it was reported that MXene microelectrodes yielded higher quality neural recordings, as the neural impedance of MXene microelectrodes (52 ± 25 kΩ) was 4-fold lower than that of the gold microelectrodes (206 ± 31 kΩ). Also, the background noise was lesser in MXene microelectrodes (3.7 ± 0.3 μVrms) compared to the gold microelectrodes (6.6 ± 2.0 μVrms) in *in vivo* neural signal recording. Additionally, in another study, MXene-based electrodes (MXtrodes) demonstrated exceptional conductivity (3015 ± 333 S/m) compared to PEDOT:PSS (7.6 ± 0.4 S/m) and rGO (0.005 ± 0.002 S/m) based composites, a wide electrochemical stability window (−1.7 to +0.6 V), and low electrode-skin impedance (0.35–0.47 kΩ cm^2^) in gel-free setups. Compared to conventional electrodes, MXtrodes enhance signal quality and allow high-resolution neural and neuromuscular mapping, effective stimulation, and artifact-free MRI/CT imaging, making them ideal for advanced bioelectronic applications [217].

In another study, Ozcan et al. developed multiwalled carbon nanotubes (MWCNTs) and Ti_3_C_2_T_X_ MXene-based electrochemical biosensors, that were able to detect amyloid-β protein, an established biomarker of Alzheimer's disease [218]. In different human plasma samples, the MXene-based biosensor was able to detect Aβ42 protein with higher sensitivity and selectivity. This sensor demonstrates a superior detection range of 1.0 fg mL⁻^1^ to 100.0 fg mL⁻^1^ and an exceptionally low limit of detection LOD of 0.3 fg mL⁻^1^ for amyloid-β protein, outperforming other sensors in this area. This highly stable, reusable biosensor represents the start of a new era in application of MXene-based biosensors for the detection of biomarkers in various neurological disorders. In summary, MXene electrodes are more sensitive, acquire information more efficiently, and show better biocompatibility, than other metal and carbon-based electrodes. Despite the limited number of studies, above examples demonstrate the potential of MXene in this field. However future studies are needed in large animal models to further demonstrate translational potential of MXene nanomaterials for development of bioelectronics for neural tissue monitoring.

#### 4.1.2 MXenes as electroactive materials in neural tissue engineering

**Fig. 4:**
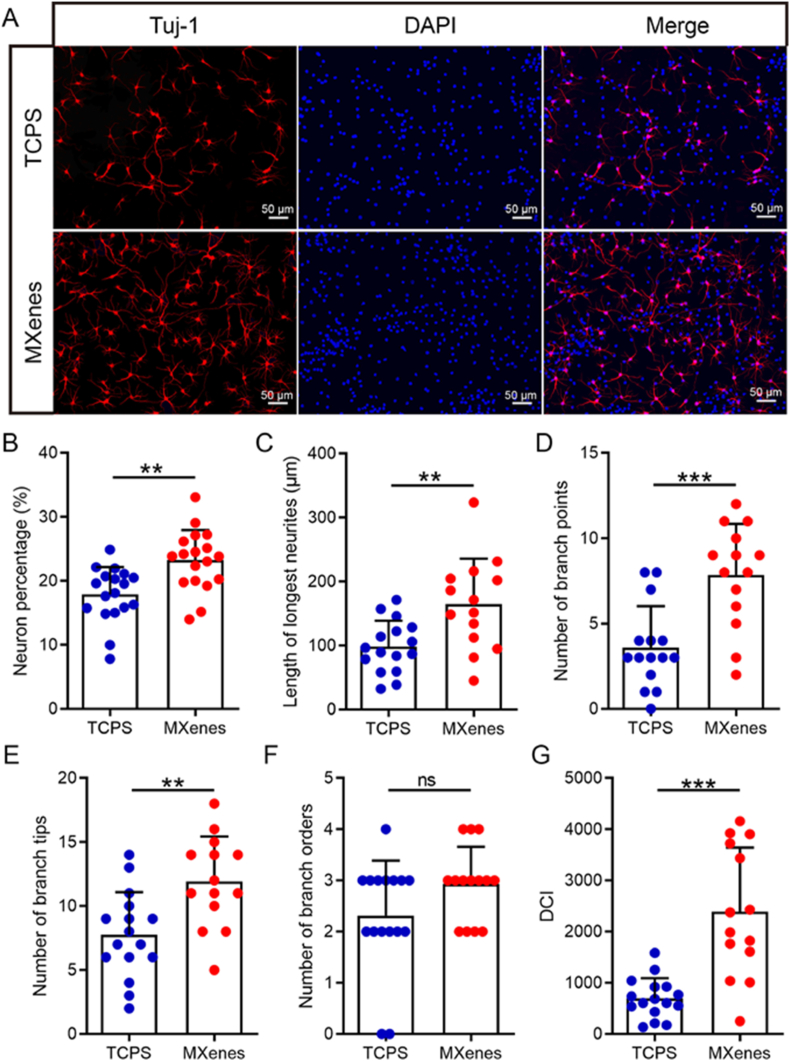
Effects of Ti_3_C_2_T_x_ MXene film on NSC differentiation. A) Representative fluorescent images of NSC progeny cultured on Ti_3_C_2_T_x_ MXene film and TCPS substrate after NSCs were induced to differentiate for 7 days. βIII-tubulin was stained red, and nuclei were stained blue. Scale bar: 50 μm. B–G) Histograms of the neuron percentage (B), the length of the longest neurites (C), the number of branch points (D), the number of branch tips (E), the number of branch order (F), and the dendritic complexity index (DCI)(G) from NSC-derived neurons. This figure is reproduced with permission from Ref. [194]. © 2020 Acta Materialia Inc. Published by Elsevier Ltd. All rights reserved.

Brain is an electrically active organ, with a complex chain of electrical, chemical and mechanical signals, that determine the homeostasis of neural cells and the brain itself. However, after an injury to the brain tissue or following a neurological disease such as Alzheimer's, there is death of functional brain cells, including neurons. Due to the complex neurophysiology and limited regenerative capacity of the damaged nervous system, restoring brain function is a big challenge in the field. To repair damaged neural networks, one of the most promising tissue engineering approaches is to create electrical microenvironments using conductive nanomaterials that mimic the neural extracellular matrix [219]. In this regard, MXenes are an excellent choice, the outstanding electromechanical properties of MXenes and flexibility of surface functionalization provides opportunities for development of ideal electroconductive substrates for neural tissue engineering. In a recent study, Guo and colleagues used Ti_3_C_2_T_x_ MXene nanosheets coated on 2D tissue culture polystyrene (TCPS) plates to investigate the impact of MXene on neural stem cells (NSCs) [194]. The Ti_3_C_2_T_x_ MXene film demonstrated high conductivity (97.1 ± 1.6 S/cm) and biocompatibility (EdU-positive cells on MXenes 18 ± 2 %), supporting NSC proliferation comparable to traditional TCPS surfaces (EdU-positive cells on TCPS 16.9 ± 0.9 %). NSCs cultured on MXene showed enhanced neural differentiation, with an increase in neuron percentage (23 ± 5 %), longer neurites (165 ± 71 μm), and more branching points (12 ± 4 μm) than those on TCPS (17.9 ± 4.3 %, 98 ± 41 μm & 8 ± 3 μm respectively) (Fig. 4). Electrical stimulation further boosted proliferation, increasing EdU-positive cells grown on MXene from 18 % to 40 % and metabolic activity (glucose consumption from 17.4 to 16.5 mmol/L; lactic acid from 1.11 to 4.1 mmol/L). MXene's conductive properties and compatibility make it promising for neural tissue engineering. Another study using MXenes in a 3D nerve guidance conduit composed of poly-L-lactic acid (PLLA) reported that presence of MXene substrate promoted cell proliferation and nerve growth [209]. The MXene/PLLA conduits significantly enhanced conductivity and neural compatibility, with conductivity increasing from 1.45 × 10^−12^ S/m (pure PLLA) to 4.53 S/m in 10 % MXene/PLLA, optimal for neural growth. PC12 cells on 10 % MXene/PLLA showed increased proliferation higher than PLLA at days 3 and 5 and longer neurite outgrowths, with most lengths ranging 10–20 μm and >20 μm by day 5. This demonstrates that 10 % MXene/PLLA provides an ideal environment for PC12 cell differentiation, with enhanced conductivity promoting neural-like morphology and cell proliferation in neural tissue engineering.

Further, in neural tissue engineering, optical properties of electrically conductive nanomaterials can be used to study the complex functions of neurons. Using a nanomaterial that boasts superior optical properties, neurons when in close contact with them will be stimulated to depolarize, without affecting the normal function of the neurons. Hence, to understand the membrane depolarization process one does not need invasive techniques, such as bioelectronic implants and genetic modifications of neurons. In this context, Wang et al. have reported the application of Ti_3_C_2_T_x_ MXene nanosheets to modulate the electrical activity of Dorsal root ganglion (DRG) neurons [220]. This study reported that non-invasive photothermal stimulation of DRGs with MXene resulted in a change in membrane potential of cells without causing any cytotoxicity. Ti_3_C_2_T_x_ MXene demonstrates strong photothermal properties ideal for neural tissue engineering. Under 808 nm NIR laser, isolated Ti_3_C_2_T_x_ flakes produced a temperature increase of 3.3 K with 10 mW, outperforming other materials like AuNRs and SiNWs. As a film (25 μg/cm^2^), MXene generated a 10.66 K rise, attributed to the cumulative effect of flakes. Therefore, both photothermally active Ti_3_C_2_T_x_ films and flakes enable effective photothermal modulation of cellular activity, requiring only low incident energy for stimulation. DRG neurons adhered well to MXene films without delamination or cytotoxicity (viability for control samples (95.4 ± 3.4 %) and MXene films (94.8 ± 3.2 %)). Photothermal stimulation on MXene films triggered Ca^2+^ transients with laser energies as low as 2 μJ (0.6 J/cm^2^), demonstrating effective neural modulation. For subcellular targeting, isolated MXene flakes allowed precise optical modulation, facilitating advanced neural stimulation at the single-cell level. Therefore, the unique physiochemical properties and electrically conductive nature of MXenes are advantageous for the creation of electroactive substrates for neural-based biomedical application.

#### 4.1.3 Influence of physicochemical properties of MXenes in neural tissue engineering

MXenes’ attributes make them the ideal candidate for developing advanced neural interfaces and scaffolds. A key advantage of MXenes is their remarkable electrical conductivity, which is crucial for neural applications where efficient signal transmission is essential [221]. Neural tissues heavily rely on electrical signals for communication, and incorporating conductive materials like MXenes into scaffolds can significantly enhance the interaction between cells and electronic devices. Notably, Ti_3_C_2_T_X_ MXenes have demonstrated the ability to facilitate electrical coupling between neurons, enabling precise monitoring and stimulation of neural activity [217]. This conductivity improvement is particularly beneficial when MXenes are integrated into bioinks for 3D bioprinting, enhancing the electrical properties of printed scaffolds to align more closely with those of natural neural tissues, thus supporting the development of bioelectronic devices, biosensors, and implants [222,223].

Beyond their conductivity, MXenes also exhibit excellent biocompatibility, making them highly suitable for neural tissue engineering. Studies have shown that MXene-coated substrates enhance the adhesion, proliferation, and differentiation of neural stem cells NSCs, which is critical for regenerating damaged neural tissues [224]. For instance, surfaces coated with Ti_3_C_2_T_X_ have been found to promote NSC neurogenesis, directing the differentiation of these cells into functional neurons and astrocytes [225]. This property is advantageous for creating scaffolds that mimic the extracellular matrix, providing an environment conducive to neural tissue repair and supporting the formation of functional neural networks essential for treating neurodegenerative diseases.

In terms of mechanical properties, MXenes exhibit high tensile strength and elasticity, making them ideal for constructing robust and flexible scaffolds that can support neural regeneration under physiological conditions. The hydrophilic nature of MXenes aids their integration into hydrogel matrices, resulting in conductive scaffolds that promote the proliferation and differentiation of NSCs, while maintaining structural integrity. These mechanical attributes ensure that MXene-based scaffolds are both resilient and capable of sustaining dynamic cellular processes, which are crucial for long-term neural tissue applications [226,227].

Furthermore, MXenes possess intrinsic antibacterial properties, which help prevent infections in implanted devices—a significant advantage for long-term implants where microbial contamination could impair functionality [228]. Future investigations into the biodegradability of MXenes should prioritize ensuring their safe degradation within biological environments, to mitigate potential risks of chronic inflammation or long-term toxicity. Moreover, optimizing the controlled biodegradation of MXene-based materials could facilitate their gradual resorption, synergizing with natural tissue healing processes and reducing the likelihood of adverse immune responses.

### 4.2 Application of MXenes for cardiac tissue engineering

#### 4.2.1 MXenes in bioelectronics for cardiac tissues

**Fig. 5:**
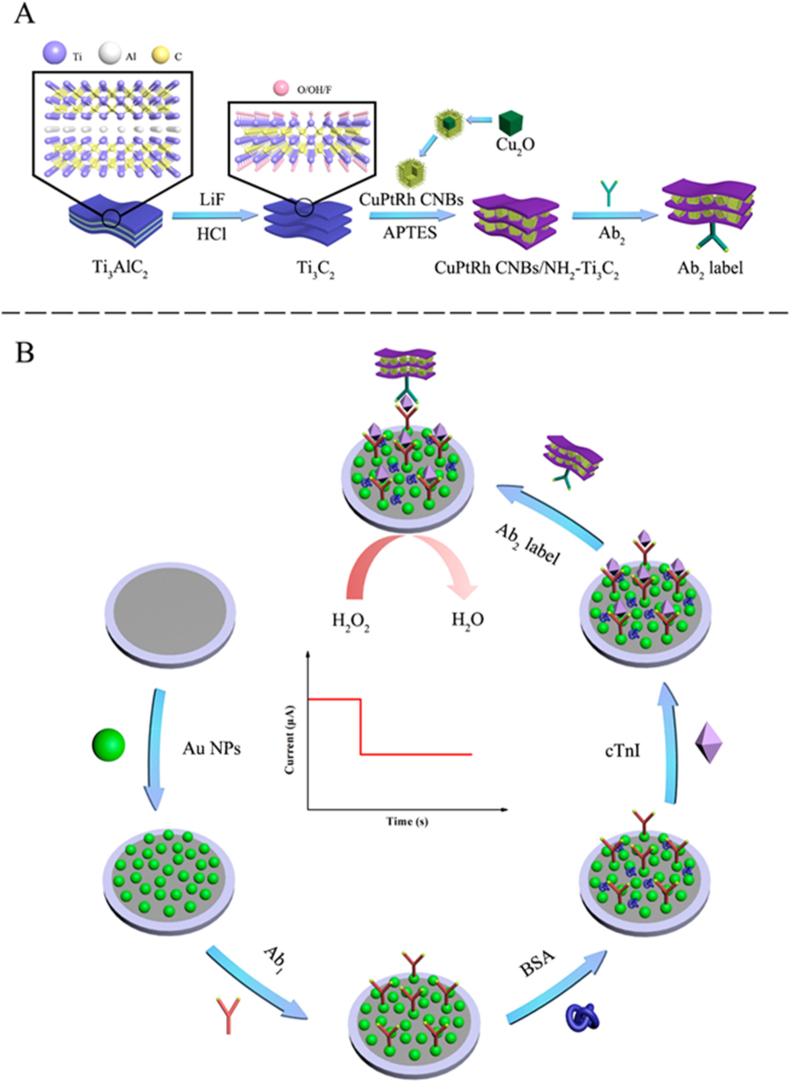
Preparation of the Ab_2_ label (A). The schemata of fabrication of the immunosensor working electrode (B). This figure is reproduced with permission from Ref. [202]. Copyright © 2020, American Chemical Society.

In cardio-bioelectronics, biosensors are promising tools for detecting cardiac biomarkers and monitoring cardiovascular disease at the onset, allowing the treatment to be initiated in a timely manner and thereby preserving the heart function. With 2D nanomaterials, biosensors can be more closely coupled with bio-analytes, which will improve cardiac biomarkers' sensitivity and robustness for rapid, accurate diagnostics of acute myocardial infarction (AMI) and other cardiac complications [229]. The electrical conductivity, large surface area, and hydrophilicity of MXenes, allow them to be well suited for cardiac sensing applications. One of the most sensitive and specific markers of AMI in patients' plasma is cardiac troponin I (CTnI). CTnI levels are around 10 ng/ml at the onset of AMI, and the levels can spike as severity increases, reaching 550 ng/ml in a short span of 18 h. Wang and colleagues developed a paper-based Ti_3_C_2_ MXene immunosensor for detecting CTnI levels [230]. The APTES-functionalized MXene sheets on paper-based screen-printing electrodes (SPEs) showed excellent conductivity and enhanced electron transfer. The immunosensor demonstrated a low LOD of 0.58 ng/mL for cTnI detection across 5–100 ng/mL, with a high linear correlation (R^2^ = 0.9745) between cTnI concentration and differential pulse voltammetry peak current reduction. Selectivity was confirmed as only cTnI significantly reduced peak current among various proteins. The sensor also showed high reproducibility, with a relative standard deviation (RSD) of 6.3 % across five sensors. In another study, to improve the sensitivity of CTnI detection, Dong et al., used trimetallic electrochemical immunosensors (CuPtRh) sandwiched with Ti_3_C_2_ MXenes and gold nanoparticles in a similar fashion as showcased in Fig. 5 [202]. This study reported that the MXene-based immunosensor demonstrated high sensitivity for cTnI detection with a broad range (25 fg/mL to 100 ng/mL) and an impressive LOD of 8.3 fg/mL (R = 0.9979). It showed reproducibility with RSDs of 2.48 % and 2.53 % for 1 ng/mL and 1 pg/mL cTnI, respectively. Selectivity was confirmed, with current fluctuations within 5 % in the presence of interferents, and stability remained at 90.94 % after 5 weeks at 4 °C. Recovery rates ranged from 99.48 % to 101.20 %, confirming the sensor's reliability and high potential for precise cTnI monitoring in cardiac diagnostics. As compared to the previous study, this electrode showed greater sensitivity and selectivity towards CTnI detection. Therefore, fabrication of an efficient biosensor with high reliability requires strong electrical conductivity and high catalytic activity for measuring cardiac biomarkers.

The other important biomarker, used for predicting coronary artery disease risk, is C-reactive protein (CRP) in the patient's serum. CRP levels in healthy adults' range between 0.5 and 10 μg/mL, and they increase with the onset of cardiovascular disease. Here in this study, Lee et al. synthesized a peptide imprinted electrochemical biosensor doped with Ti_2_C MXenes to detect CRP levels in serum [231]. MXene-doped peptide-imprinted polymer (pRIP/Ti_2_C) electrodes demonstrated enhanced electrochemical sensing for cardiac biomarkers. Compared to non-imprinted (NIP) electrodes, pRIP/Ti_2_C showed improved redox response with an extended sensing range of 0.1–10,000 fg/mL and an LOD of 0.2 ag/mL for CRP. The current density increase was 1.1–1.3 times higher due to Ti_2_C doping stating that MXene integration amplifies electron transfer efficiency and enhances signal sensitivity. Stability tests indicated reusability across five cycles with minimal performance loss, and AC impedance showed a stable charge transfer resistance around 3 Ω. This MXene-enhanced sensor offers high sensitivity, a broad detection range, and durability for precise cardiac diagnostics.

In addition to probing important cardiac biomarkers, MXenes have also been used in wearable technology to fabricate biocompatible, high-performance flexible pressure sensors that can measure heart rate and monitor heart health [232]. The silk fibroin SF@MXene biocomposite film-based sensor demonstrated excellent flexibility and durability, with an effective elastic modulus reduced to 1.22 MPa from 2.11 MPa for pure MXene. The sensor exhibits high sensitivity across two pressure ranges: 25.5 kPa^−1^ (100–500 Pa) and 3.4 kPa^−1^ (1–20 kPa) compared to pristine MXene. With a fast response/recovery time of 40/35 ms and robust performance over 3500 cycles at 73 Pa, it is ideal for continuous monitoring. Applied on the wrist, the sensor detects subtle heart rate pulses, distinguishing diastolic and percussion waves, showing its potential for cardiac biomonitoring. Its array configuration enables spatial mapping, making it versatile for wearable health monitoring and human-computer interaction. In summary, MXene-based biosensors offer a high surface-to-volume ratio, compact size, and distinctive electrical and optical properties, making them excellent candidates for use in cardiovascular disease diagnosis and as sensing materials.

#### 4.2.2 MXenes as electroactive materials for cardiac tissue engineering

**Fig. 6:**
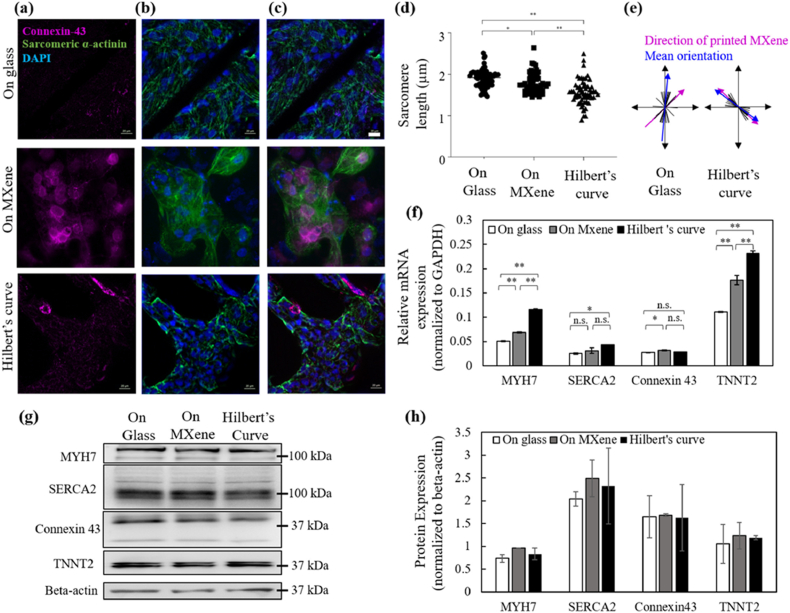
Immunostaining characterization and qRT-PCR analysis of iPSC derived cardiomyocytes at day 7 (All scale bars indicate 20 μm). (a) The connexin 43 (CX-43) staining of the straight line-patterned Ti_3_C_2_T_*x*_MXene printed on glass controls, on unpatterned Ti_3_C_2_T_*x*_ MXene controls, and Hilbert's curve-patterned Ti_3_C_2_T_*x*_ MXene printed on PEG. (b) The sarcomeric alpha-actinin staining of on-glass controls, on-Ti_3_C_2_T_*x*_*MXene* controls and Hilbert's curve. Cell nuclei are stained with DAPI (blue). (c) Combined Connexin-43 and sarcomeric alpha-actinin staining of on-glass controls, on- Ti_3_C_2_T_*x*_MXene controls and Hilbert's curve. Cell nuclei are stained with DAPI (blue). (d) Sarcomere length quantification in μm (∗ represents *p* < 0.05 and ∗∗ represents *p* < 0.01). (e) Directionality analysis. (f) qRT-PCR analysis of relative mRNA expression of cardiac markersMYH7, *SERCA2*, GJA1, and TNNT2 (∗∗ represents *p* < 0.01 and n.s. represents non-significant) (g) Western blotting for the expressions of MYH7, SERCA2, GJA1*,* and TNNT2 proteins. (h) Quantification for the western blotting (*n* = 2). This figure is reproduced with permission from Ref. [200]. © 2020 Acta Materialia Inc. Published by Elsevier Ltd. All rights reserved.

After a myocardial infarction (MI) there is a blockade in the coronary artery that leads to lack of blood supply to the heart muscle and death of functional cardiomyocytes. Since cardiomyocytes are terminally differentiated therefore heart muscle has a limited capacity to regenerate itself, even after normal blood circulation is restored to the heart. Therefore, left ventricular dysfunction following myocardial infarction ultimately leads to heart failure. The conventional medical therapies are able to provide temporary relief to the condition. However, cardiac tissue engineering-based strategies have the potential to offer permanent solution to the problem by replacing damaged myocardium with functional cardiac tissues. In this regard several studies have reported development of biomaterials-based cell laden scaffolds which mimic the native myocardium. These scaffolds after implantation in the injured heart have been able to preserve cardiac function [[233], [234], [235], [236], [237], [238]]. In recent studies, electroconductive nanomaterials have been used to improve the electromechanical properties of cardiac scaffolds and patches. However, they have not yet been able to fully replicate the native electroconductive myocardium. In this regard, MXenes are employed, due to their high surface area, flexibility for surface functionalization and electrical properties*.* Ye and colleagues reported for the first time the synthesis and application of Ti_2_C MXenes in cardiac tissue engineering after an MI [239]*.* The Ti_2_C-doped cryogel significantly enhanced mechanical strength (from 2.24 to 9.65 kPa), conductivity (around 0.1 S/m) and elasticity (10.13 kPa), matching the natural myocardium's properties. Cardiomyocytes (CMs) cultured on Ti_2_C-8-cryogel showed >90 % viability and well-formed sarcomeres, with strong Ca^2^⁺ signaling and three-fold higher contraction amplitude than Ti_2_C-free cryogels. In an MI rat model, Ti_2_C-8-cryogel ECPs improved cardiac function, elevating ejection fraction (EF) and fractional shortening (FS) compared to controls. This MXene-enhanced cryogel demonstrates superior conductivity, elasticity, and biocompatibility, promoting synchronized CM beating and myocardial repair. In literature different methods have been used to fabricate electroconductive composites that match the native ECM of the human heart. One such method is to 3D print biocompatible and biomimetic cardiac patches, that contain conductive nanomaterials to improve the material characteristics of the construct. In a study by Basara et al., a conductive cardiac patch was created using 3D printed Ti_3_C_2_T_x_ MXenes embedded in a PEG hydrogel [200]. The Ti_3_C_2_T_x_ MXene-PEG hydrogels demonstrated significant advancements in cardiac tissue engineering by promoting superior alignment and maturation of iCMs. Cell viability was consistently high, starting at over 85 % by day 2 and increasing to 93 % by day 7. The introduction of patterned MXene surfaces resulted in enhanced sarcomere alignment (6.3° deviation compared to 34.9° on glass), which is crucial for proper cardiac function. Additionally, gene expression for key markers like MYH7 (2.3-fold), TNNT2 (2.1-fold), and SERCA2 (1.7-fold) was significantly upregulated, indicating improved maturation (Fig. 6). These enhancements translated into optimized Ca^2+^ signal propagation, with conduction velocities of 4.3–6.5 cm/s between regions, facilitating synchronized contractions. Notably, the beating cell area on Hilbert-patterned MXene was substantially larger (82 %) compared to glass (36 %). These findings underscore the potential of MXene-PEG hydrogels to improve biocompatibility, conductivity, and functional cellular organization, positioning them as a promising material for cardiac tissue repair and regeneration*.* These studies demonstrate that MXene-based nanomaterials have the potential for cardiac tissue repair after an injury.

Recently, the capability to develop electrospun MXene-loaded materials with potential for cardiac tissue regeneration were developed. Here, polycaprolactone (PCL), a slow-degradable and highly biocompatible polymer, that is widely used in tissue engineering, was employed To overcome the hydrophobic nature of PCL and facilitate MXene deposition, various post-treatment methods were implemented, including alkaline and acidic treatments [240,241], as well as oxygen plasma treatment [242]. Using the electrospinning technique followed by MXene deposition via dip coating, highly porous 3D scaffold, suitable for cardiac tissue regeneration, was obtained. MXenes were tightly deposited on PCL fibers (Fig. 7.1), penetrating deeply into the 3D PCL electrospun network (Fig. 7.2). The conductivity of the PCL-MXene composite ranged from 5.22 mS/m to 326.33 mS/m (Fig. 7.3), which is suitable for cardiac tissue engineering, as the conductivity of normal myocardium is approximately 1 mS/m. Further, it was demonstrated that, irrespective of the post-treatment methods (chemical or oxygen plasma), PCL-MXene materials remained biocompatible during 7 days of cell cultivation. On day 7, a uniform and aligned distribution of fibroblasts (Fig. 7.4) was observed across the surface of both uncoated and MXene-loaded PCL membranes. At high magnification, tight cell-cell contacts resembling syncytium were observed. Thus, it was hypothesized that the application of the PCL-MXene membrane to damaged heart tissue could enhance the conduction of electrical signals from healthy tissue to ischemic areas via MXenes, thereby improving the contractile function of the heart.

**Fig. 7:**
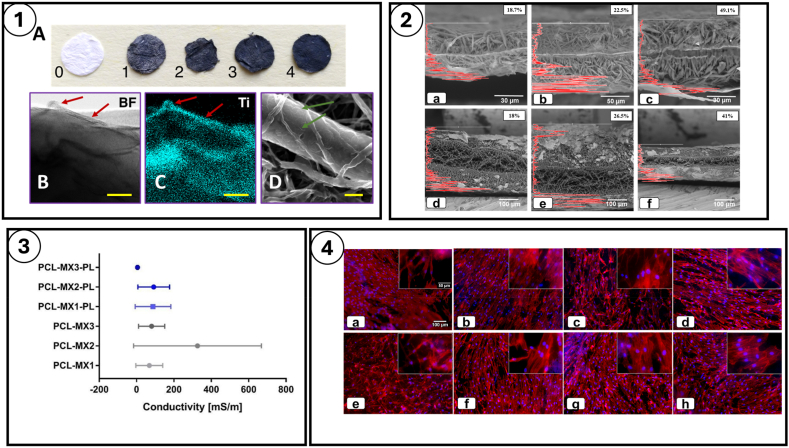
**(7.1)** - PCL electrospun nanofiber membranes with immobilized MXene (A) with the visualization of MXene flakes on the surface of PCL fibers – B (bright field TEM), C (EDX map) and D (SEM image). This figure is reproduced with permission from Ref. [241]. Copyright © 2023, American Chemical Society. **(7.2)** - Scanning electron microscopy images of membranes' cross-section with cross-sectional EDX line scan (red diagrams) and EDS atomic % of Ti (a-f represent different deposition modes) with **(7.3)**- recalculated conductivity of MXene coated PCL membranes and **(7.4)** - Fluorescent images of nuclei (blue) and cytoskeleton staining (red) on the 7th day of dermal fibroblasts on PCL-membranes with various numbers of deposited layers (a–h). This figure is reproduced with permission from Ref. [242]. Copyright © 2023, The Author(s), under exclusive license to Springer Nature Switzerland AG.

Nevertheless, additional research is required to gain a deeper understanding of the underlying mechanisms, which will be crucial in developing strategies for successfully translating these therapies into clinical practice for patient care.

#### 4.2.3 Influence of physicochemical properties of MXenes in cardiac tissue engineering

The physiochemical properties of MXenes position them as ideal candidates for developing scaffolds that closely mimic the native cardiac environment, thereby enhancing cardiac tissue repair and regeneration. A critical advantage of MXenes in cardiac applications is their exceptional electrical conductivity, which is crucial for ensuring efficient signal transmission between cardiomyocytes. This electrical coupling supports synchronized contractions, which are essential for the functional maturation of engineered cardiac tissues [243,244]. In addition, the mechanical properties of MXenes further enhance their utility in cardiac tissue engineering, as they provide the necessary strength and elasticity to support the dynamic mechanical environment of the heart. MXene-based hydrogels, for instance, have been developed to mimic the elasticity of native cardiac tissues, offering mechanical support to damaged regions and promoting tissue repair which were discussed in the above sections. Additionally, MXenes exhibit remarkable versatility in their integration with various biomaterials, such as hydrogels, cryogels, and electrospun fibers, enabling the design of multifunctional scaffolds tailored to specific cardiac tissue engineering needs. This versatility, combined with modifiable surface chemistry, enhances cell adhesion, proliferation, and differentiation, making MXenes adaptable for diverse tissue engineering applications.

Beyond their electrical and mechanical advantages, MXenes possess antioxidant properties that help mitigate oxidative stress—a common issue following myocardial infarction. By reducing oxidative damage, MXenes protect cardiomyocytes from further injury and promote tissue regeneration. Also, MXenes exhibit excellent biocompatibility, interacting seamlessly with biological systems and minimizing the risk of immune reactions. This characteristic is vital for their integration into cardiac tissues without triggering inflammation or rejection [36,245].Their intrinsic antibacterial activity also reduces the risk of infections associated with implanted devices, making them highly suitable for biomedical applications.

### 4.3 Application of MXenes in bone repair and regeneration

**Fig. 8:**
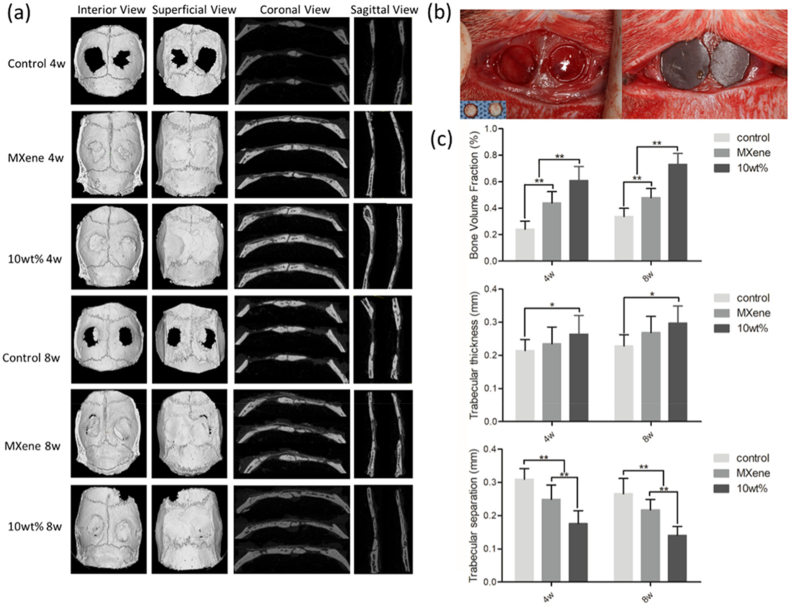
Bone regeneration performance in control group, MXene group and UHAPNWs/MXene group at 4 and 8 weeks after surgery: (a) three-dimensional reconstruction images of bone defect including interior and superficial views and corresponding micro-CT images including coronal and sagittal views. (b) Surgical process to build rat calvarial defect model and cover the bone defect with relevant membranes. (c) Radiographic analysis of bone morphometric parameters, including Bone volume fraction (BV/TV, %), trabecular thickness (Tb. Th, mm), and trabecular separation (Tb.Sp, mm), ∗*p* < 0.05, ∗∗*p* < 0.01 compared with indicated groups. This figure is reproduced with permission from Ref. [210]. © 2020 Elsevier B.V. All rights reserved.

In the recent years bone substitute materials are of great interest for bone reconstruction to negate insufficient bone volume and poor bone tissue regeneration following an assault to bone. The next generation biomaterials have revolutionized the field of bone regeneration and repair [246]. Some of the biomaterials such as graphene and its derivatives have been widely explored for application as scaffolds, hydrogels and guided bone regeneration membranes [247]. However, MXene nanomaterials have shown promise in bone regeneration and repair because of their exceptional physiochemical properties, including mechanical and electrical characteristics. Based on their similarity to the native microenvironment of bone tissues, MXene nanosheets facilitate the support of cell attachment, growth, differentiation, and calcium binding properties. Native bone and periosteum have an electrical potential that contributes to their volume and quality [248,249]. By design, MXenes have inherent electrical properties that mimic physiological and electrical characteristics of bone tissue, making them promising candidates for bone tissue regeneration. Reports from a recent study suggest that hydroxyapatite ultralong nanowires (UHAPNWs)/MXene nanocomposite membranes have the ability to recapitulate the micro/nano architecture of native bone microenvironment and exhibit both osteo-inductivity and osteo-conductivity promoting bone formation [210]. In rat calvarial defect model, incorporation of UHAPNWs into MXene presented a relatively increased trabecular thickness and increased quality of regenerated bone tissue compared to control group which can be observed in Fig. 8 [210]. Also, in another study, Jiebing et al., reported that pre-osteoblastic cells grown on multilayered Ti_3_C_2_T_x_ MXene films exhibited a wider cytoplasmic extension, multiple filopodial attachments and enhanced osteogenic differentiation by upregulating the mRNA levels of ALP, OPN and OCN. *In vivo* implantation of MXene films to rat model of calvarial defects demonstrated high bone formation, early osteogenesis and quicker biomineralization compared to clinically used material titanium mesh [205]. These reports emphasize on the fact that MXene nanomaterials can be the ideal candidate for bone repair and regeneration.

However, it is also reported that with time, the bonds between the layers of MXene sheets start deteriorating and disintegrating that questions the stability of naïve MXenes. Therefore, mixing of MXene nanomaterials with suitable polymers to prevent it from degrading could provide better strategies for bone regeneration [205]. In this regard, Ti_3_C_2_ MXene incorporated sodium alginate/hydroxyapatite 3D printed scaffolds were highly stable which exhibited high mechanical strength, electrical conductivity and excellent biocompatibility. The presence of MXene sheets supported initial cell attachment, spreading with prominent filopodium, enhanced ALP activity at initial stages of osteogenesis and matrix mineralization after 14 days promoting late osteogenesis *in vitro* [250]. Similarly, in another study, the addition of OTES-Ti_3_C_2_T_z_ nanosheets significantly improved the hydrophilicity, mechanical rigidity and electrical conductivity of PLA membranes thereby enhancing MC3T3-E1 pre-osteoblasts cell adhesion and osteogenic differentiation [98]. Just like PLA, PCL is another hydrophobic polymer and incorporating MXene into the electrospun PCL nanofibers significantly enhanced its hydrophilicity and conductivity which improved biomineralization, protein adsorption and cell attachment in this study [206].

The conventional treatments for bone repair following massive defects due to surgical removal of osteosarcoma involve implanting orthopedic components/materials, but the outcome is mostly not favourable due to the presence of tumor microenvironment. As a result, under these conditions smart materials such as MXenes have found their way into bone repair because these materials exhibit excellent osteo-conductivity, osteo-inductivity, and tumor ablation properties. In this regard, niobium carbide (Nb_2_C) MXene nanosheets incorporated into bioglass ceramic scaffolds (NBGS) showed anti-cancer properties by reducing the viability of SaoS2 cells. The NBGS effectively restrained the growth of osteosarcoma in a mouse model without causing any other pathological complications indicating its biosafety [199]. Surprisingly, it also promoted vasculogenesis of endothelial cells *in vitro* and stimulated angiogenesis *in vivo* supporting the formation of new bone tissue via osteogenesis and angiogenesis***.*** In another study, the postoperative tissue lesion following osteosarcoma excision was effectively cured by a multifunctional hydrogel containing MXene nanosheets, bioinert sulfonated polyether ketone, gelatin methacrylate (GelMA) and tobramycin. The presence of MXene promoted photothermal ablation of tumor and osteogenic properties of the construct [211]. Also, in another study a photothermal bone scaffold containing MXene, collagen (col), hydroxyapatite and silk showed effective killing of squamous CAL-27 cancer cells *in vitro* and inhibited tumor growth under *in vivo* conditions [251]. Therefore, treatment strategies such as the above studies have the potential to reduce off target effects of chemotherapeutic strategies in killing cancer cells or ablating tumor growth.

The photothermal property of MXene is not only utilized for killing osteosarcoma cells, the same was also explored for accelerated release of bioactive ions from the osteogenic biomaterial. In this regard, a nanofiber system with hydroxyapatite nanoparticles (HA) and MXene nanosheets, upon NIR (808 nm) irradiation promoted an enhanced osteogenic differentiation owing to the accelerated release of Ca, P and Mg ions from HA [212]. These unique properties of MXene highlight its potential for development of multifunctional biomaterial systems capable of releasing bioactive ions in-response to irradiation and promote repair process. Overall, corroborating these reports, it is evident that MXenes possess excellent potential for bone repair and regeneration however, further investigations are needed to understand the mechanisms of MXene mediated beneficial effects.

### 4.4 MXenes for artificial muscles

**Fig. 9:**
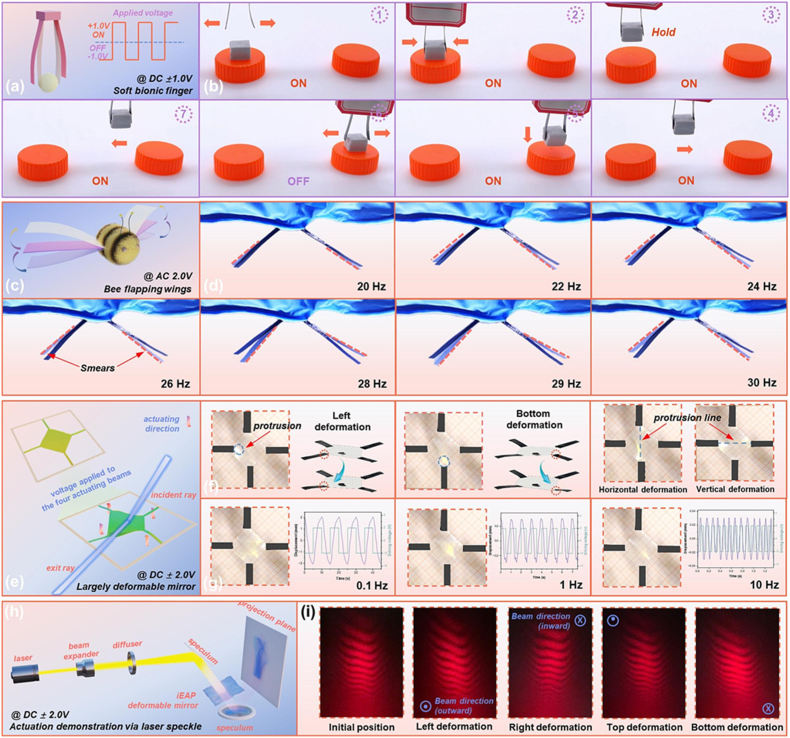
Soft robotic applications of the BC-IL-MXene electro-ionic actuators. (a–b) The demonstration of grapple robot consisting of soft bionic fingers for object loading under DC 1.0 V. (c–d) The demonstration of high-frequency bioinspired flapping wings under AC 2.0 V at frequency of 20–30 Hz. (e–g) The demonstration of 3D-adaptive motion actuating platform of deformable mirror in terms of static and dynamic performance. (h) The optical configuration and (i) its magnified projection of laser speckles for actuation demonstration of iEAP-based adaptive platform of beam-plate deformable mirror. This figure is reproduced with permission from Ref. [256]. © 2023 Elsevier B.V. All rights reserved.

The skeletal muscles are the type of muscles that connect to different bones in the body and facilitate a wide range of voluntary movements and functions. Inspired by the functioning of skeletal muscles inside the body, significant efforts are being made by different research groups to fabricate biomimetic materials that can regulate the movements of robots. In this regard, several natural as well as synthetic electroactive materials, including carbon nanotubes and graphene are being widely explored for their application in the fabrication of artificial muscle fibres [252]. These artificial muscle materials coiled into yarns are capable of inducing axial contraction in response to stimulus-induced radial expansion by unwinding of the coiled yarns. The major drawback of this muscle system during the entire actuation cycle is the zero net work done (i.e. the amount of weight the muscle is able to lift is needed for the muscles to return to its initial contracted state upon stimulus removal) [253]. To solve this challenge muscles with an elongation mode of working are being developed with small or no load needed for return actuation. Also, a self-sensing property of muscle fibers is essential for constant muscle movement monitoring. To solve these challenges, Lizhing et al., developed a self-sensing Ti_3_C_2_T_x_ MXene/SWCNTs coated coaxial muscle fibre composite which has the capability of bi-lengthwise actuation in response to heat and solvent adsorption. MXene/SWCNTs layers were able to exert a piezoresistive effect providing real-time, self-position sensing ability to the fibers warranting its use as artificial muscles in bionic arm [254]. The artificial muscles also require longer durability, bending strain and quick response. In a separate study, Ti_3_C_2_T_x_ ionically cross-linked with poly (3,4-ethylenedioxythiophene)-poly(styrenesulfonate) demonstrated a rapid response time of less than 1 s and showcased outstanding cyclic stability [255]. Similarly in another study [256], low-voltage electro-ionic soft actuators with large deformation strain, suitable for applications in artificial muscles, flexible electronics, and soft robotics, are enhanced here through a nanocomposite membrane incorporating ionically crosslinked bacterial cellulose (BC) nanofibers and Ti_3_C_2_T_x_ MXene nanomaterials as shown in the graphical abstract in Fig. 9. This 3D interpenetrating structure improves ion selectivity and transport by reducing energy barriers, yielding actuators with 0.88 % bending strain, 10.8 mm tip displacement, ultra-stable cycling, and a broad response up to 18 Hz at 0.5 V, demonstrating potential for adaptive 3D motion systems in advanced soft robotics. These unique properties widen the scope of MXenes for application in the manufacturing of next generation robotic systems.

Overall, the available literature suggests that MXenes have excellent potential in the field of robotics, prosthetics (wearable devices), and development of organ on a chip platform mimicking native human tissues for drug development. However, more research is required to understand the mechanisms and evaluate long term safety of MXene-based materials in small as well as large animal models. These initiatives would help in development of therapeutic products and clinical translation of these technologies.

### 4.5 Application of MXenes in skin tissue engineering

Health care providers face many challenges when it comes to treating wounds resulting from trauma, diabetes, and burns, which pose a challenge to their ability to treat patients [[257], [258], [259]]. In order to heal wounds, drugs, skin grafting, and cellular treatments are employed to speed up the healing process. However, as a therapeutic method, some of these treatments have limitations, which prevent their effectiveness and make them impractical in some cases [[260], [261], [262]]. MXenes have been studied as an alternative therapy due to their distinct properties in different areas of biomedical science. Therefore, MXenes have attracted researchers in the area of wound healing. In particular, the high photothermal conversion efficiency, excellent conductivity, large surface area, and antibacterial properties of MXenes make them excellent candidates for application in skin tissue engineering. A study by Mao et al. used Ti_3_C_2_T_x_ as conductive nanomaterials in combination with regenerated bacterial cellulose (rBC) to develop multifunctional hydrogels that stimulate wound healing by modulating the behavior of skin cells. Compared to other composites, the 2 wt% MXene/rBC composite exhibited the highest electrical conductivity, desirable mechanical properties, and very good flexibility. In addition, MXene containing hydrogels are reported to accelerate wound healing in NIH3T3 cells when combined with electrical stimulation [263].

**Fig. 10:**
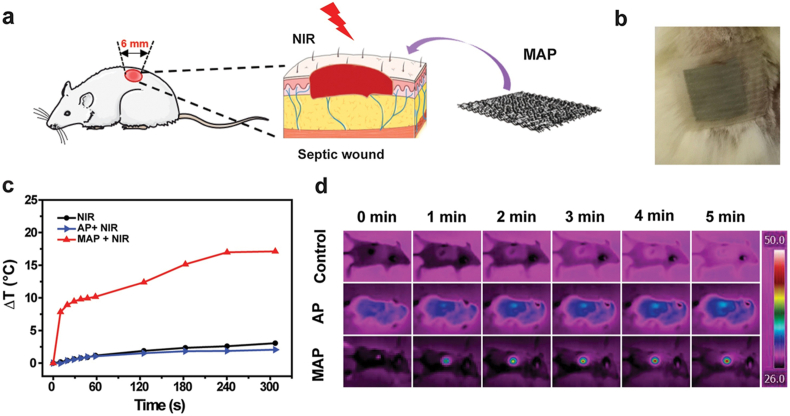
(a) A model of S. aureus-infected Balb/c mice; (b) images of the MAP nanofibrous membrane serving as an antibacterial dressing; (c) temperature increase of the mice with S. aureus-infected wounds; (d) corresponding thermal images of (c). This figure is reproduced with permission from Ref. [265]. © 2021 Elsevier B.V. All rights reserved.

Wound healing takes place in three primary stages: inflammation, proliferation, and remodeling. Throughout these phases, the body engages in a continuous and intricate repair process that can be hindered by infection. Therefore, the use of antibacterial agents to prevent and treat infections is crucial for promoting faster wound healing. Ti_3_C_2_T_x_ MXene has shown significant antibacterial effectiveness against *Escherichia coli* and *Bacillus subtilis*, making it a promising material for this purpose [264]. In another study, a multimodal nanofibrous composite was prepared by adding amoxicillin (AMX) to Ti_3_C_2_T_x_ MXene in PVA for treatment of wound infection as shown in Fig. 10. The photothermal property of MXene facilitated the effective release of AMX which had prolonged antibacterial activity against *S. aureus* and promoted wound healing process *in vivo* [265]. As a result of the above studies, it is evident that MXenes have a broad range of applications in the field of skin tissue engineering.

## 5 Challenges and Prospective advances for MXenes in clinical applications

While MXenes hold considerable promise in biomedical applications, particularly in tissue engineering and regenerative medicine, several critical challenges must be addressed before their clinical translation can be realized [286]. These challenges include concerns about biosafety, stability, morphology control, and functionalization, among others. Below, we outline the main limitations associated with MXenes in clinical research, offering a comprehensive review of the obstacles hindering their widespread application.

### 5.1 Biosafety and biocompatibility

Ensuring the long-term biosafety of MXenes remains a significant challenge. Although they are generally considered biocompatible based on initial studies, their long-term effects within biological systems are not fully understood. This lack of long-term safety data is particularly concerning for clinical applications, where materials will remain in contact with biological tissues for extended periods [287]. Further, *in vivo* studies are needed to fully assess the potential for adverse reactions, including inflammation, immune responses, or toxicity, particularly in sensitive applications such as cardiac and neural tissue engineering. The potential cytotoxicity of MXenes, especially at higher concentrations, is another critical concern. While lower concentrations of MXenes have demonstrated good cytocompatibility, higher doses may lead to cellular toxicity, affecting tissue viability and function. This necessitates careful optimization of MXene concentrations in tissue engineering applications to balance efficacy with safety [288]. Developing standardized protocols for determining safe dosage ranges will be crucial for advancing MXenes in clinical settings.

### 5.2 Stability and degradation

Stability in physiological environments is a major obstacle for MXenes. They are prone to degradation when exposed to body fluids, which can limit their utility in long-term biomedical applications. For instance, the release of incorporated drugs or bioactive agents from MXene-based systems must be carefully controlled to ensure consistent therapeutic effects over time. However, current studies have shown that achieving a stable, sustained release profile remains challenging. Moreover, the tendency of MXenes to undergo oxidation in physiological conditions complicates their long-term use in clinical applications [289]. This oxidation may lead to a loss of electrical conductivity, negatively affecting their performance in electroactive tissue engineering applications, such as cardiac or neural scaffolds. The degradation behavior of MXenes *in vivo* also requires a more comprehensive understanding to prevent potential adverse effects from the byproducts of degradation.

### 5.3 Morphology and structural control

The morphology and structural properties of MXene-based scaffolds are vital for their effective application in tissue engineering. Controlling the morphology—such as achieving a uniform dispersion of MXenes within composite materials—is essential for ensuring the mechanical strength, biocompatibility, and cellular interactions of these scaffolds [290]. However, achieving uniform dispersion and integration of MXenes into polymer matrices, such as polycaprolactone (PCL), presents a significant challenge. Variations in the structural composition can lead to inconsistent mechanical properties, affecting scaffold performance and cellular responses. Moreover, the integration of MXenes into polymer matrices requires precise control over the fabrication process to ensure the desired structural and functional properties. This level of control is critical for applications that demand specific mechanical and electrical characteristics, such as scaffolds for electroactive tissues. A lack of uniformity in the structural properties of MXene-based scaffolds may result in suboptimal outcomes, including poor cell adhesion, limited differentiation, and inadequate tissue regeneration.

### 5.4 Targeting and functionalization

Enhancing the targeting capacity of MXene-based biomaterials for tissue-specific applications is another area that requires further research. For MXenes to be effective in regenerative medicine, they must be able to interact specifically with the target tissues, such as cardiac, neural, or skeletal tissues. This involves improving their surface modifications to enhance their ability to bind to or be taken up by specific cell types [291]. Currently, the surface functionalization of MXenes to optimize their bioactivity and therapeutic potential in complex tissue environments is still in its early stages. Functionalization strategies for MXenes must be refined to ensure they can adequately support cellular signaling and tissue regeneration. For example, the integration of bioactive molecules, such as growth factors or peptides, onto the surface of MXenes could enhance their ability to promote cell differentiation and tissue repair. However, these strategies must be carefully tailored to avoid unintended effects, such as cytotoxicity or an immune response. Moreover, the synthesis, dispersion, and surface chemistry functionalization of MXenes are complicated processes that require precise control to achieve the desired outcomes.

### 5.5 Scalability

Their scalability for clinical use presents several challenges that must be addressed. One major issue is environmental instability. MXenes are highly prone to oxidation and degradation, particularly in humid environments, which severely limits their shelf life and operational reliability in industrial settings. This instability necessitates controlled storage conditions and handling procedures, complicating large-scale production and increasing operational costs [292]. While strategies like incorporating antioxidants, low-temperature storage, and thermal annealing can improve stability, they introduce additional steps to an already complex production process, further hindering scalability.

Another key challenge lies in synthesis methods. Conventional synthesis of MXenes often relies on hazardous chemicals such as hydrofluoric acid (HF), which raises significant safety and environmental concerns. The use of HF not only complicates large-scale production due to stringent safety measures and waste management protocols but also limits the environmental friendliness of MXene production [293]. Although alternative, fluoride-free etching methods are being explored to mitigate these risks, these techniques are still in development and require further optimization to be viable for industrial-scale production.

In addition, intrinsic material properties pose further obstacles to scalability. MXenes tend to restack due to their two-dimensional structure, which reduces the accessible surface area and consequently diminishes their performance. Surface modifications and the development of heterostructures with other materials have been proposed to address this issue, but these approaches also add complexity and cost to the production process.

### 5.6 Limitations in tissue-specific applications

While MXenes have shown potential in various tissue engineering applications, there are limitations that hinder their effectiveness in specific tissues. For example, their osteogenic activity and suitability for bone tissue engineering remain unclear. Although MXenes have been investigated for their potential in bone regeneration, particularly in guided bone regeneration therapies, the understanding of their interactions with bone tissue is still limited. The mechanical strength of MXene-based composites must be optimized to match the load-bearing requirements of bone. Additionally, their bioactivity needs to be enhanced to support osteogenesis and mineralization, while minimizing potential cytotoxicity due to metal ion release during degradation. Further studies are needed to clarify the role of MXenes in promoting osteogenic differentiation and supporting bone tissue repair.

Similarly, the use of MXenes in cardiac tissue engineering presents challenges. Despite their excellent electrical conductivity, MXene-based materials have not yet achieved the level of success needed for the complete repair of cardiac tissues, such as those damaged by myocardial infarction. The complexity of the myocardial microenvironment, coupled with the need for precise electrical and mechanical properties, makes it difficult to replicate the conditions necessary for effective cardiac regeneration. Also, MXenes must withstand the highly dynamic and oxidative environment of the myocardium. Their susceptibility to oxidation and potential structural degradation could impair their electrical properties and integration with cardiac cells. Ensuring consistent mechanical compatibility without eliciting arrhythmic or adverse immune responses is another critical challenge.

While MXenes offer excellent electrical conductivity and flexibility for neural tissue repair, concerns regarding their long-term stability and potential neurotoxicity remain. The interaction between MXenes and neural cells over extended periods, particularly in terms of inflammatory responses and degradation byproducts, needs further investigation. In addition, the poor differentiation efficiency of transplanted neural stem cells remains a significant obstacle in using MXenes for neural tissue engineering. Effective substrates are required to promote the growth and differentiation of NSCs, but the current understanding of how MXenes support these processes is limited.

Furthermore, the flexibility of surface modification through functionalization and combining MXenes with other materials to synthesize need based composites creates a promising scope for these wonder materials in the field of tissue engineering. However, for clinical translation of MXene-based products for diagnosis and disease management of electroactive tissues, future studies should be directed toward understanding the underlying mechanisms of MXene-mediated biomedical effects. It is critical to understand how MXenes behave with cells at protein and gene levels. Extensive *in vivo* studies are required to understand the long-term safety of MXenes, and comparative studies with other biomaterials such as graphene, TMP, and h-BN are essential for advancing MXenes-based products in the near future.

## 6 Conclusion

In summary, while MXenes offer significant potential in tissue engineering and regenerative medicine, several challenges must be overcome before their widespread clinical use. These include addressing concerns about biosafety, stability, structural control, functionalization and scalability. Future research should focus on improving the long-term stability of MXenes in physiological environments, enhancing their targeting and bioactivity through surface modifications, and ensuring their safe and effective use across various tissue types. Only by addressing these limitations can MXenes realize their full potential in clinical applications, particularly in the fields of electroactive tissue engineering.

## CRediT authorship contribution statement

**Keshav Narayan Alagarsamy:** Writing – review & editing, Writing – original draft, Investigation, Formal analysis, Data curation, Conceptualization. **Leena Regi Saleth:** Writing – original draft, Methodology, Investigation, Formal analysis, Data curation. **Saravanan Sekaran:** Methodology, Investigation, Formal analysis, Data curation, Conceptualization. **Laura Fusco:** Methodology, Investigation, Formal analysis, Data curation. **Lucia Gemma Delogu:** Investigation, Funding acquisition, Formal analysis, Data curation. **Maksym Pogorielov:** Investigation, Formal analysis, Data curation, Conceptualization. **Açelya Yilmazer:** Investigation, Formal analysis, Data curation, Conceptualization. **Sanjiv Dhingra:** Writing – review & editing, Validation, Supervision, Resources, Project administration, Funding acquisition, Data curation, Conceptualization.

## Declaration of competing interest

The authors declare that they have no known competing financial interests or personal relationships that could have appeared to influence the work reported in this paper.
